# High numbers of activated helper T cells are associated with better clinical outcome in early stage vulvar cancer, irrespective of HPV or p53 status

**DOI:** 10.1186/s40425-019-0712-z

**Published:** 2019-09-03

**Authors:** Kim E. Kortekaas, Saskia J. Santegoets, Ziena Abdulrahman, Vanessa J. van Ham, Marij van der Tol, Ilina Ehsan, Helena C. van Doorn, Tjalling Bosse, Mariëtte I. E. van Poelgeest, Sjoerd H. van der Burg

**Affiliations:** 1 Department of Gynecology, Oncode Institute, Leiden University Medical Centre, PO Box 9600, 2300 RC Leiden, The Netherlands; 2 Department of Gynecology, Leiden University Medical Centre, PO Box 9600, 2300 RC Leiden, The Netherlands; 3 Department of Medical Oncology, Oncode Institute, Leiden University Medical Centre, PO Box 9600, 2300 RC Leiden, The Netherlands; 4 Department of Gynecologic Oncology, Erasmus MC Cancer Institute, University Medical Center Rotterdam, PO Box 2040, 23000 CA Rotterdam, The Netherlands; 5 Department of Pathology, Leiden University Medical Centre, PO Box 9600, 2300 RC Leiden, the Netherlands

**Keywords:** Vulvar cancer, Tumor microenvironment, Immunotherapy, T cells, PD-1

## Abstract

**Background:**

Vulvar squamous cell carcinoma (VSCC) has been suggested to consist of three subtypes; HPV-positive, HPV-negative mutated *TP53* or HPV-negative *TP53* wildtype, with different clinical courses. To analyze the immune infiltrate in these molecular subtypes and its impact on clinical outcome, an in-depth study of the tumor immune microenvironment was performed.

**Methods:**

Sixty-five patients with invasive VSCC matched for age, FIGO stage and treatment modality, were grouped according to the presence of HPV and p53 protein expression status. Archived tissues were analyzed for intraepithelial and stromal expression of CD3, CD8, Foxp3, PD-1, and pan-keratin in randomly selected areas using immunofluorescence. Additional phenotyping of T cells was performed ex-vivo on VSCC (*n* = 14) and blood samples by flow cytometry. Healthy vulvar samples and blood served as controls.

**Results:**

Based on T-cell infiltration patterns about half of the VSCC were classified as inflamed or altered-excluded while one-third was immune-deserted. High intraepithelial helper T cell infiltration was observed in 78% of the HPV-induced VSCC, 60% of the HPVnegVSCC/p53wildtype and 40% of the HPVnegVSCC with abnormal p53 expression. A high intraepithelial infiltration with activated (CD3^+^PD-1^+^), specifically helper T cells (CD3^+^CD8^−^Foxp3^−^), was associated with a longer recurrence-free period and overall survival, irrespective of HPV and p53 status. Flow cytometry confirmed the tumor-specific presence of activated (CD4^+^PD-1^++^CD161^−^CD38^+^HLA-DR^+^ and CD8^+^CD103^+^CD161^−^NKG2A^+/−^PD1^++^CD38^++^HLA-DR^+^) effector memory T cells.

**Conclusion:**

This is the first study demonstrating an association between intraepithelial T cells and clinical outcome in VSCC. Our data suggest that abnormal p53 expressing VSCCs mostly are cold tumors whereas HPV-driven VSCCs are strongly T-cell infiltrated.

**Electronic supplementary material:**

The online version of this article (10.1186/s40425-019-0712-z) contains supplementary material, which is available to authorized users.

## Introduction

Immunotherapy of cancer has established itself as a new breakthrough approach that offers long-term durable clinical responses in patients with advanced cancers. As the initiation and regulation of the immune response to tumors is complex and multistep in nature, inspection of the different processes involved is required to provide the optimal (combination) of immunotherapeutic modalities available [1]. This is highly relevant for vulvar squamous cell carcinoma (VSCC), the predominant histologic subtype of vulvar cancers, for which new treatment options are urgently needed. Because despite current treatment, consisting of radical surgery and/or (chemo) radiotherapy which causes impressive morbidity, lymphedema, sexual and psychological dysfunction and wound healing disorders [2, 3], 46% of VSCC patients still develop recurrences after 10-years [4].

At this point however, little is known about the role and impact of cellular immunity on the clinical outcome of VSCC. Both CD4 and CD8 T cells as well as B cells infiltrate VSCC [5–7]. The CD4 cells comprise CD4^+^ helper T cells and regulatory T cells (Tregs). Often a strong infiltration with one type of T cells is paralleled by the others [5, 6, 8]. In three studies focussing on the prognostic role of CD4^+^ and/or CD8^+^ T cells or Tregs no impact on clinical outcome was found [6, 9, 10]. On the one hand, these analyses may have been influenced by the heterogeneity of the study group with respect to tumor etiology, stage and treatment. Furthermore, enumeration of all T cells, irrespective of their location in the tumor [9], as well as preselection of highly infiltrated areas only [6, 10], may also have influenced study outcomes. On the other hand, the impact of T cells may be nullified by the presence of immune regulatory mechanisms, as VSCC can be massively infiltrated with M2 macrophages and Tregs [8]. Moreover, VSCC can express the immunoregulatory enzyme, indoleamine 2,3-dioxygenase (IDO) or PD-L1, both of which were shown to negatively influence clinical outcome [10, 11]. Notably, PD-L1 was mainly expressed in lymphocyte rich areas [11], suggesting that it functioned as an adaptive escape mechanism [12], and implying that in some VSCC a functionally active antitumor response is present. This notion is sustained by the observation that the intraepithelial presence of Granzyme B-positive cells is related to better overall survival (OS) in patients with localized VSCC [13].

At present, three distinct etiologic pathways in the development of VSCC have been proposed. One type is driven by the overexpression of high-risk human papilloma virus oncogenes E6 and E7 (HPVposVSCC). The second type is not related to HPV and can be categorized based on the mutational status of the tumor suppressor gene *TP53* associated with high protein levels of p53 (HPVnegVSCC/p53abn). We recently identified a third type as a substantial group of patients with a HPV-negative VSCC displaying normal expression levels of p53 protein (HPVnegVSCC/p53wt) but frequently bearing other mutations [14]. Importantly, HPV-driven VSCC display better OS and a longer recurrence-free period (RFP) than HPVnegVSCC [14–17]. Interestingly among the latter group, local recurrences more often occurred after treatment in patients with HPVnegVSCC/p53abn than in HPVnegVSCC/p53wt [14]. With the first reports showing an influence of different oncogenic pathways on local immunity [18, 19], we asked the question if the differences in RFP and OS observed between the three groups of VSCC driven by different oncogenic pathways could be explained by the local immune response. Bearing in mind the limitations of previous studies, we selected three cohorts of VSCCs based on their HPV and p53 protein (abn/wt) status which were highly matched for clinicopathological parameters and enumerated different types of intraepithelial and stromal T cells in randomly selected fields of VSCC, using multiplex immunofluorescence. In-depth analysis of T cells was performed on freshly dispersed tissue by flow cytometry. Our study revealed a strong impact of intraepithelial activated T cells on clinical outcome, in particular a dense infiltration with intraepithelial CD4^+^ T cells was highly associated with RFP and OS, irrespective of HPV or p53 status. Moreover, the percentage of tumors highly infiltrated with these T cells varied between the three different subtypes, with HPV-induced VSCC most often strongly infiltrated (78%) followed by the HPVnegVSCC/p53wt (60%) and the lowest infiltration in the HPVnegVSCC/p53abn group (40%).

## Material and methods

### Patient materials

Archived formalin-fixed paraffin-embedded (FFPE) tumor tissue from VSCC patients was selected from a larger cohort with known HPV and p53 status. HPV presence was tested by HPV-PCR and p16 IHC [20]. Tumors that were positive in both tests were assigned as HPVposVSCC. When both tests were negative, tumors were scored as HPVnegVSCC. The HPVnegVSCC were further sub-classified based on the wildtype or abnormal expression of p53 (HPVnegVSCC/p53wt and HPVnegVSCC/p53abn) as previously described [14]. In addition, archived FFPE healthy HPV-negative vulvar tissue from 10 women who underwent labial reduction surgery served as controls. Fresh tumor tissue (*n* = 14) and blood samples (*n* = 34) were obtained from 38 patients participating in the large observational CIRCLE study. Women with histologically proven p16^ink4a^-negative VSCC were included in this study investigating cellular immunity against anogenital lesions [21, 22]. Tumor staging was done according to FIGO staging 2009. Patients were included after signing informed consent. The study was conducted in accordance with the Declaration of Helsinki and approved by the local medical ethical committee of the Leiden University Medical Center (P08.197 and B16.024) and in agreement with the Dutch law. The materials were used according to the Dutch Federation of Medical Research Association guidelines. The patients received standard-of-care treatment consisting of primary surgery.

### Multiplex six color staining, image acquisition and analysis by VECTRA

For the direct and indirect immunofluorescent six color staining and detection, 4 μm FFPE tissue sections were deparaffinized and prepared with heat-induced antigen citrate (10 mM, pH 6.0) retrieval as described previously [23]. Antibody specificity and optimal conditions for antigen retrieval were assessed by single-plex IHC using tonsils as a positive control [23]. After incubation with superblock buffer (Thermo Fisher Scientific, Waltham, MA, USA), the primary antibodies and isotype/species-specific secondary fluorescent antibodies were applied (Additional file 1). Nuclear counterstain was obtained with DAPI. Tissue slides were imaged at 20x magnification with the Vectra 3.0 Automated Quantitative Pathology Imaging System (Perkin Elmer). Imaging analysis and spectral separation of dyes was performed with the InForm Cell Analysis software (Perkin Elmer) by using spectral libraries defined with single-marker immunofluorescence detection. Five random multispectral imaging fields of interest were selected for acquisition from each tumor, depending on its size. Tissue and cellular segmentation was done as described before [23]. The following phenotypes were identified for the T cell panel: total T cells (CD3^+^), CD8^+^ T cells (CD3^+^CD8^+^Foxp3^−^), helper T cells (CD3^+^CD8^−^Foxp3^−^), Tregs (CD3^+^CD8^−^Foxp3^+^), PD-1 expressing T cells (CD3^+^PD1^+^). All images were visually inspected to confirm the correct attribution and quantification of phenotypes, and segmentation of tissue. Because PD-1 could be expressed by CD3^+^CD8^−^ and CD3^+^CD8^+^ cells, the CD3^+^PD1^+^ phenotypes were separately analyzed. All phenotypes in both areas were normalized by tissue area (number of cells/mm^2^). In addition, ten HPVposVSCC, six HPVnegVSCC/p53wt and five HPVnegVSCC/p53abn VSCC samples were used to study Tbet (Santa Cruz, clone H-210, dilution 1:100) expressing CD3^+^ cells with immunofluorescence.

### Blood and tumor cell isolation and culturing

Venous blood samples were drawn prior to surgery, and peripheral blood mononuclear cells (PBMC) were isolated using Ficoll density gradient centrifugation as described previously [24, 25]. VSCC tumor material was obtained and handled as described [24, 25]. First, tumor material was cut into small pieces. One-third of the tumor pieces was incubated for 60 min at 37 °C in Iscove’s Modified Dulbecco’s Medium (IMDM, Gibco by life technologies, ThermoFisher Scientific, Lonza, Verviers, Belgium) with 10% human AB serum (Capricorn Scientific, Esdorfergrund, Germany) and supplemented with high dose of antibiotics (50 μg/ml Gentamycin (Gibco/ Thermo Fisher Scientific (TFS), Bleiswijk, the Netherlands), 25 μg/ml Fungizone (Gibco/Thermo Fisher Scientific), after which the tumor pieces were put in culture in IMDM supplemented with 10% human AB serum (IMDM complete) and 1000 IU/ml human recombinant IL-2 (Aldesleukin, Novartis, Arnhem, the Netherlands). Cultures (*n* = 14) were replenished every 2–3 days with fresh IMDM complete and IL-2 to a final concentration of 1000 IU/ml. After 2–4 weeks, when sufficient T cells were obtained, the cells were cryopreserved and stored in liquid nitrogen until use. Approximately two-third of the tumor pieces was incubated for 15 min at 37 °C in IMDM dissociation mixture containing 10% human AB serum, high dose of antibiotics (as above) and 0.38 mg/ml of the commercially available Liberase enzymes (Liberase TL, research grade, Roche). Following incubation, cell suspension was put on a 70 μm cell strainer (Falcon, Durham, NC, USA) to obtain a single cell suspension, counted using trypan blue exclusion (Sigma, St Louis, MO, USA), and cryopreserved at approximately 2 million cells/vial. All cells were stored in the vapor phase of liquid nitrogen until further use.

### Flow cytometry and data analysis

Cryopreserved PBMC (*n* = 34) and/or cryopreserved freshly isolated single cell tumor samples (*n* = 12) were thawed and assessed by flow cytometry as described before [26, 27]. In brief, samples were thawed according to standard operation procedures and stained with the LIVE-DEAD® Fixable yellow dead cell stain kit (ThermoFisher Scientific) for 20 min at room temperature to identify dead cells. Following incubation, the cells were washed, incubated with PBS/0.5%BSA/10%FCS for 10 min on ice to block Fc receptors. After the cells were washed, the cells were stained for 30 min on ice and in the dark with fluorochrome-conjugated antibodies. Intracytoplasmic/intranuclear staining was conducted with the BD Pharmingen Transcription Factor Buffer set (BD Biosciences) according to manufacturers’ protocol. Details on antibodies used are listed in Additional file 1. Acquisition of cells was done on a BD LSR Fortessa. Data was analyzed by either manual gating using DIVA software (version 8.02; BD Biosciences) or by high-dimensional single cell data analysis using hierarchical Stochastical Neighbor Embedding (HSNE) [28] in Cytosplore. To automatically discover stratifying biological signatures at the single cell level, we used the fully automated hierarchical clustering (unsupervised) tool CITRUS in the cloud-based cytobank software (Fluidigm Sciences) with an FDR of 1%.

### Cytokine production of phytohemagglutinin (PHA)-stimulated TIL

To determine capacity of tumor infiltrating lymphocyte (TIL) batches from HPVnegVSCC tumors to produce cytokines in response to mitogenic stimulation, cultured TIL batches (*n* = 14) were stimulated with 0.5 μg/ml PHA (HA16 Remel; ThermoFischer Scientific) for 4 days, after which supernatants were harvested and analyzed by Cytometric Bead Array (CBA, Th1/Th2 kit, BD Bioscience, Breda, the Netherlands) according to the manufacturer’s instructions. The cut-off value for cytokine production was 20 pg/ml, except for IFN-γ for which it was 100 pg/ml. Positive cytokine production was defined as at least twice above that of the unstimulated cells [25, 29].

### Statistical analysis

For data analysis the statistical software package SPSS 23.0 (SPSS Inc., Chicago, IL) was used. Group comparisons of categorical data were performed by chi-square test. The non-parametric Mann-Whitney U test was used for continuous variables when comparing two groups. For the survival analysis, patients were categorized into two groups based on numerical immune cell count. First, the median cell count was used as cut-off value. To optimize the chance to detect a relationship between T-cell subsets and clinical outcome in a relatively small group of patients, the best cut-off value for the different T cell subsets was determined using receiver operating characteristics (ROC) curve analysis. The T-cell subset values with the best accuracy (i.e. with greatest sensitivity and specificity) were selected as the most optimal cut-off value for (OS or RFP). Based on these cut-off values, the immune cell counts were categorized in two groups and a log-rank test was performed to calculate the difference in OS or RFP. The RFP was censored for lost-to-follow up and death. Two sided *p*-values < 0.05 were considered significant. GraphPad Prism 7 (GraphPad Software Inc., LA Jolla, CA, USA) was used to illustrate the data by graphs and figures.

## Results

### Patient cohort

A cohort of 65 primary VSCC samples, divided in HPVposVSCC (*n* = 23), HPVnegVSCC/p53wt (*n* = 20), and HPVnegVSCC/p53abn (*n* = 22) was analyzed. All cases were matched for age (40–85 years), FIGO stage, absence of lymph node and distant metastasis, ≥8 mm tumor-free margins, no use of immunosuppression, and no documented medical history. However, HPVposVSCC were younger than the other groups despite matching because younger women are more likely to have HPVposVSCC than HPVnegVSCC [30]. An overview of patient characteristics and treatment is given in Additional file 2. In line with current literature [15, 17, 31], the group of patients with HPVposVSCC displayed a better OS and RFP than those with HPVnegVSCC (Additional file 3). Furthermore, the recurrence rate increased from HPVposVSCC (13%), HPVnegVSCC/p53wt (40%) to 59% in HPVnegVSCC/p53abn **(**Additional file 2**).** Together this confirms our selection of a representative cohort of patients for our study.

### The HPVposVSCCs are most often strongly infiltrated with T cells

**Fig. 1:**
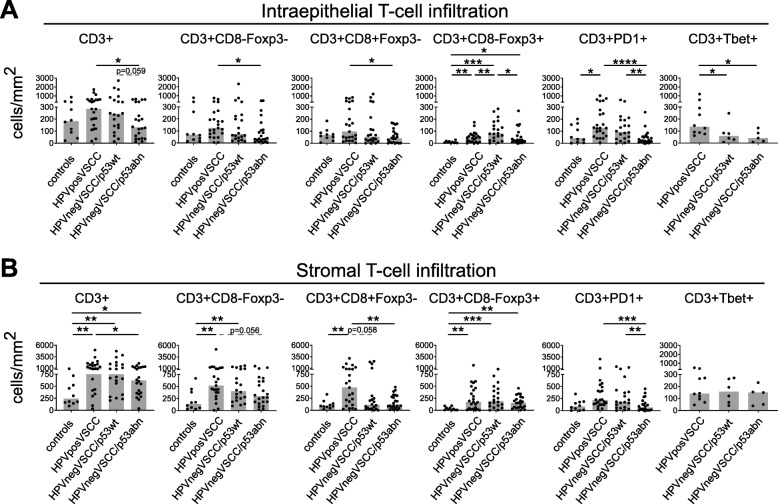
HPVposVSCC are highly infiltrated with CD3+ T cells, especially CD3^+^CD8^−^Foxp3^−^ and CD3^+^CD8^+^Foxp3^−^ cells. The numbers of intraepithelial (**a**) and stromal (**b**) infiltrating CD3 (T cells), CD3^+^CD8^−^Foxp3^−^ (helper T cells), CD3^+^CD8^+^Foxp3^−^ (cytotoxic T cells), CD3^+^CD8^−^Foxp3^+^ (regulatory T cells) and CD3^+^PD1^+^ T cells as cells/mm^2^ for HPV-negative healthy labia (*n* = 10), and HPVposVSCC (*n* = 23), HPVnegVSCC/p53wt (*n* = 20) and HPVnegVSCC/p53abn (*n* = 22) patients. CD3^+^Tbet^+^ T cells were counted on a subset of the total cohort of 10 HPVposVSCC, 6 HPVnegVSCC/p53wt and 5 HPVnegVSCC/p53abn. VSCC categorization was based on HPV-PCR, p16 and p53 IHC as described in materials and methods. The bars indicate the median cell count, individual samples are indicated by closed circles. Differences between two groups were calculated with a Mann-Whitney test with the significance indicated with asterisks. (*p < 0.05, **p < 0.01, ***p < 0.001, and ****p < 0.0001)

The archived tissues sections were simultaneously analyzed for the expression of CD3, CD8, Foxp3, PD-1, and pan-keratin by multispectral immunofluorescence VECTRA analysis, both in the epithelial and stromal compartments **(**Additional file 4). Quantification of the T cells per square mm of tumor revealed that the stroma of VSCC was more densely infiltrated with CD3^+^ T cells, CD3^+^CD8^−^Foxp3^−^ T cells, CD3^+^CD8^−^Foxp3^+^ Tregs, and CD3^+^CD8^+^Foxp3^−^ T cells than healthy controls. The number of intraepithelial Tregs was also higher in VSCC (Fig. 1**;** Additional file 4**;** Additional file 5). Comparison of the three subgroups revealed a strong difference in T cell infiltration between HPVposVSCC and HPVnegVSCC/p53abn. The majority of HPVposVSCC was well infiltrated whereas the HPVnegVSCC/p53abn most often displayed a low T-cell infiltration. The group of HPVnegVSCC/p53wt showed a more variable pattern, with low and high T-cell infiltrated tumors (Fig. 1**;** Additional file 4**;** Additional file 5). The number of tumor-infiltrating intraepithelial cells was highly correlated to the other intraepithelial T-cell subsets and to their numbers in the stroma (Additional file 6). This suggests a coordinated response of CD3^+^CD8^−^Foxp3^−^ and CD3^+^CD8^+^Foxp3^−^ T cells in VSCC. Quantification of intraepithelial CD3^+^Tbet^+^ T cells, representing type 1 immunity [27], revealed higher numbers in HPVposVSCC compared to both HPVnegVSCC subtypes (Fig. 1**;** Additional file 5).

### Immune inflamed, altered-excluded, altered-immunosuppressed and deserted VSCC

**Fig. 2:**
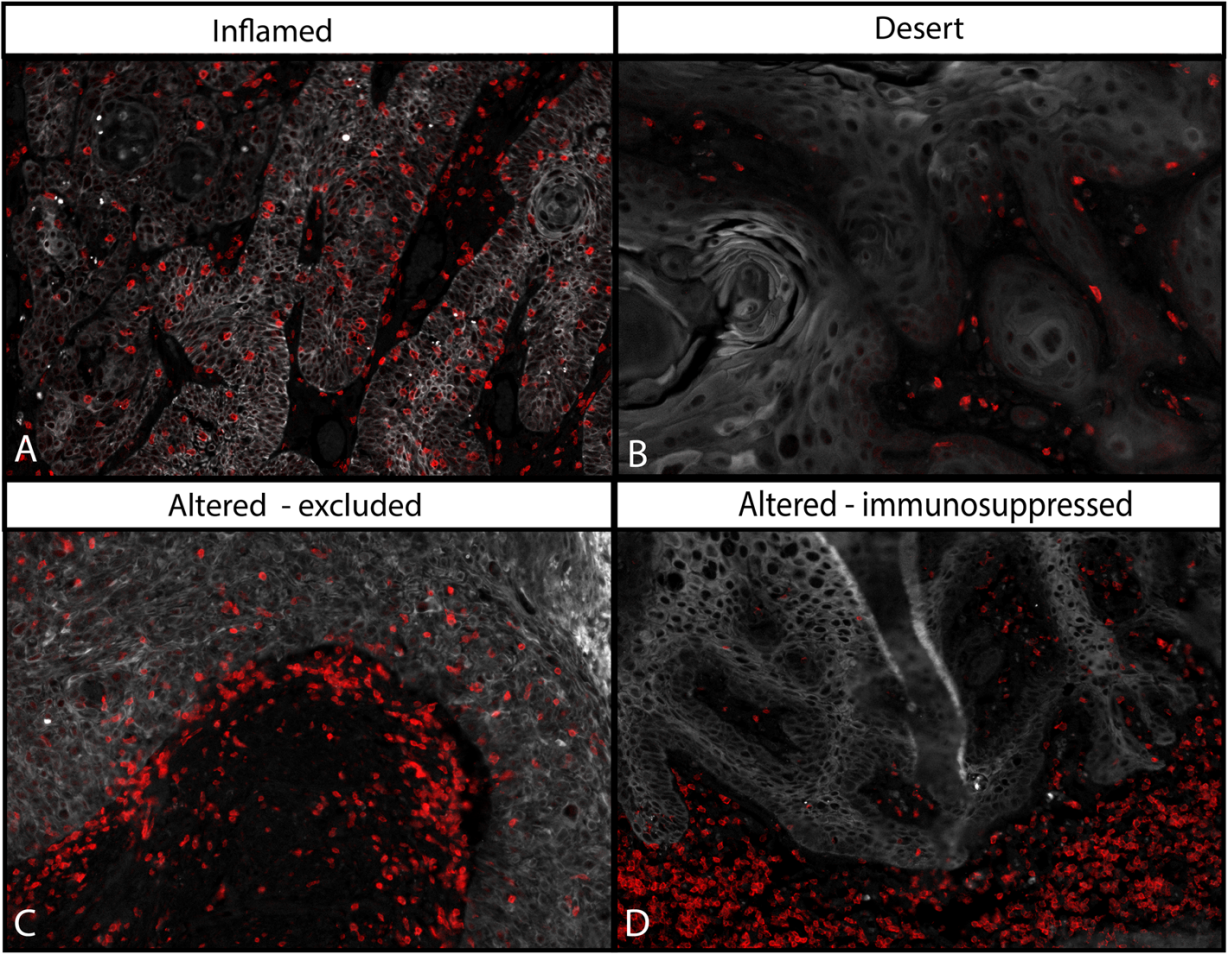
The T-cell infiltration pattern can be used to classify VSCC into four categories. Categorization of the VSCC based on the pattern of T-cell infiltration was done according to literature [32, 33]. Depicted are four representative examples of T-cell infiltration patterns: designated immune-inflamed (**a**), −deserted (**b**), −altered excluded (**c**) and -altered immunosuppressed (**d**). Altered-excluded tumors show more T cells at the invasive border rather than in the middle of the stroma. Red = CD3, white = keratin (epithelial area)

Based on the previously published categories of T-cell infiltration patterns [32], the VSCC were characterized (Fig. 2) as immune-deserted (*n* = 19), −altered (*n* = 41) or -inflamed (*n* = 5). The immune-altered group was the largest and could be subdivided based on two distinct patterns of T cells in the stroma [33]. The altered-excluded tumors (*n* = 24) showed more stromal T cells at the invasive border whereas in the altered-immunosuppressed VSCC (*n* = 17) T cells were dispersed throughout the whole stroma (Fig. 2). Notably, the number of CD3^+^ T cells at the invasive border (Fig. 2c) was highly correlated with the intraepithelial CD3^+^ T-cell count (*p* = 0.000; Additional file 6) in the altered-excluded VSCC. Moreover, the average number of intraepithelial CD3^+^ T cells in the altered-excluded was higher than in the altered-immune suppressed (mean 612 ± SD 539 vs mean 157 ± SD 92, *p* < 0.001, respectively). To evaluate the impact of these four VSCC categories on survival, a Kaplan-Meier analysis was performed. The immune-inflamed group displayed a superior RFP and OS **(**Additional file 7**)**. Interestingly, the group of altered-excluded VSCC showed a similar OS whereas the RFP was less good when compared to that of the immune-inflamed group. Therefore, the immune inflamed and altered-excluded VSCC were classified as hot tumors.

### The intratumoral CD3^+^CD8^−^Foxp3^−^ T cell count is an independent prognostic marker for RFP and OS irrespective of VSCC type

**Fig. 3:**
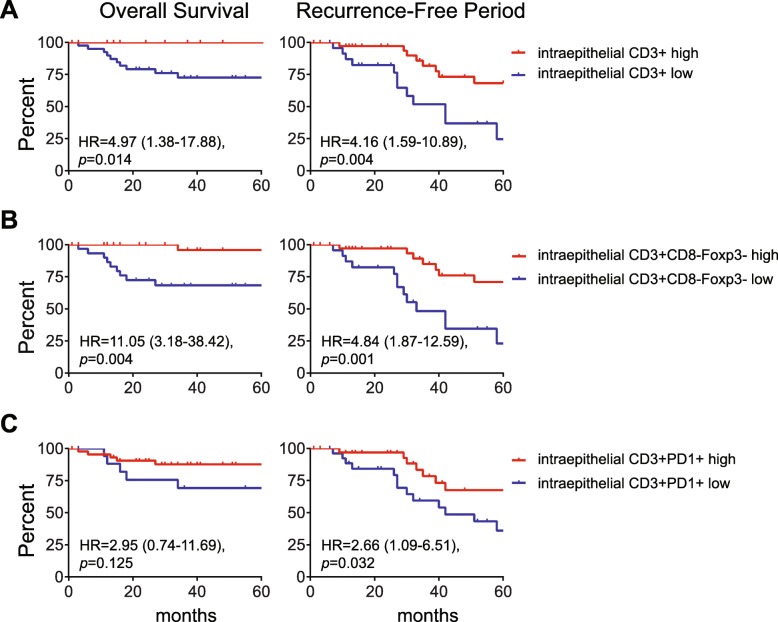
High numbers of intraepithelial CD3+ and CD3+CD8-Foxp3- T cells are associated with longer overall survival and recurrence-free period. Kaplan-Meier curves showing overall survival (left) and the recurrence-free period (RFP; right) for VSCC patients with high (red) and low (blue) numbers of intraepithelial CD3^+^ (**a**) and CD3^+^CD8^−^Foxp3^−^ (**b**) and CD3^+^PD1^+^ (**c**) cells/mm^2^. The patients were grouped based on the best cut-off value for each subset as determined by receiver operating characteristics (ROC) curve analysis. The most accurate T-cell subset values for either OS or RFP was used. Cut-off values were for CD3^+^ T cells 309.4 and 192.7 cells/mm^2^ for OS and RFP, respectively, and for CD3^+^CD8^−^Foxp3^−^ T 82.58 and 61.82 cells/mm^2^, respectively, and for CD3^+^PD1^+^ 37.67 (OS) and 99.96 (RFP) cells/mm^2^ for CD3^+^PD1^+^ cells, respectively. Patients with a T-cell count < cut-off value were classified as low, the others as high. Statistical significance of the survival distribution was analyzed by log-rank testing and significant differences were indicated with an asterisk (*p < 0.05, **p < 0.01, *** *p* < 0.001 and **** *p* < 0.0001)

The better OS of the two categories displaying stronger intraepithelial infiltration than altered-immune suppressed and immune-desert VSCC suggested an important role for intraepithelial T cells on clinical outcome. For each T-cell subset the median cell count **(**Additional file 8**)** and the optimized cut-off point, as determined by ROC curve analysis, was used to categorize the patients tumor into low or high infiltrated and subsequently its impact on clinical outcome was determined. High intraepithelial infiltration with CD3^+^ T cells, in particular of CD3^+^CD8^−^Foxp3^−^ T cells was strongly associated with longer RFP (*p* = 0.001) and OS (*p* = 0.004). A strong infiltration with CD3^+^PD1^+^ T cells was also associated with a longer RFP (*p* = 0.032). The intraepithelial infiltration with CD3^+^CD8^+^ T cells or CD3^+^CD8^−^Foxp3^+^ Tregs was not prognostic for clinical outcome **(**Fig. 3**;** Additional file 9**)**. Importantly, the prognostic power of CD3^+^CD8^−^Foxp3^−^ T cells for RFP was retained when only the HPVnegVSCC were analyzed **(**Additional file 9**)**. To sustain this notion, the impact of tumor infiltrating CD3^+^CD8^−^Foxp3^−^ T cells in clinical outcome was corrected for age, and p53 and HPV status **(**Additional file 10**)**. In the univariate analysis, only high CD3^+^CD8^−^Foxp3^−^ counts and age correlated with RFP. In the multivariate analysis, high infiltration with CD3^+^CD8^−^Foxp3^−^ T cells but not age was associated with longer RFP (HR 3.30 (1.22–8.94), *p* = 0.018). Thus, the CD3^+^CD8^−^Foxp3^−^ T-cell infiltration is expected to be an important prognostic marker for clinical outcome, irrespective of whether these VSCC are caused by the HPV-derived oncogenes or other oncogenic pathways (e.g. p53 mutation).

### HPVnegVSCC are infiltrated by activated CD8^+^ and CD4^+^ effector memory T cells

**Fig. 4:**
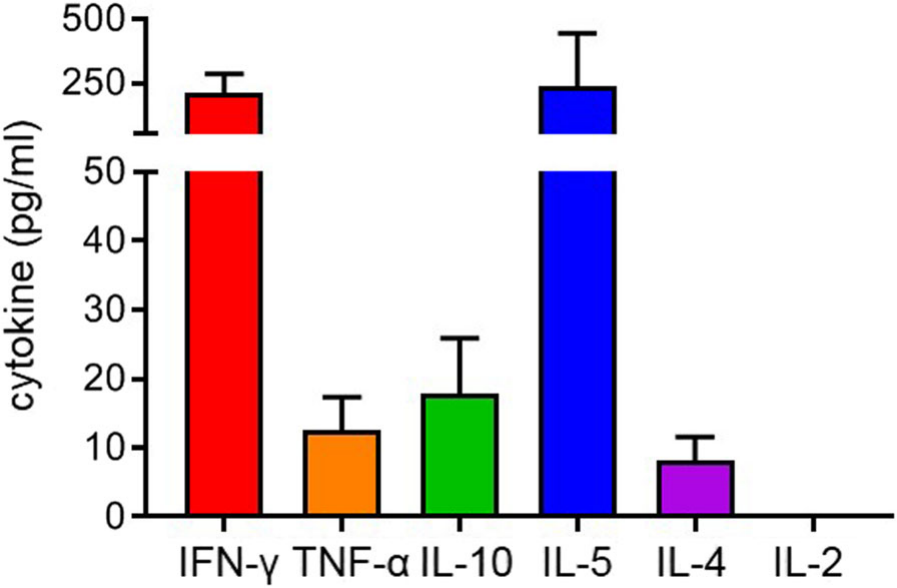
Tumor-infiltrating lymphocytes produce Th1 and Th2 cytokines upon mitogenic stimulation. In vitro expanded T cells from VSCC were analyzed for their cytokine production following mitogenic stimulation with 0.5 μg/ml PHA for 4 days, after which supernatants were harvested and analyzed by cytometric bead array (CBA) to determine the production of IFN-γ, TNF-α, IL-10, IL-5, IL-4 and IL-2 in pg/mL. Mean (± SEM) cytokine production is shown for 14 HPVnegVSCC

The vast majority (~ 80%) of vulvar cancers are not induced by HPV. [17] While 78% (18/23) of HPVposVSCC were strongly infiltrated with CD3^+^CD8^−^Foxp3^−^ T cells, there was also a considerable fraction of HPVnegVSCC displaying evidence of their immunogenicity with 60% (12/20) of the HPVnegVSCC/p53wt and 40% (9/22) of the HPVnegVSCC/p53abn showing high intraepithelial CD3^+^CD8^−^Foxp3^−^ T-cell infiltration and longer RFP and OS. In order to gain a better understanding of these tumor-infiltrating T cells in HPVnegVSCC, a serie of fresh HPVnegVSCC tumor biopsies was used to culture tumor-infiltrating lymphocytes (TIL; *n* = 14) and for ex-vivo phenotypic analysis of freshly dissociated and directly liquid nitrogen stored tumor-infiltrating T cells by flow cytometry (*n* = 12). Upon mitogenic stimulation, the growing TILs predominantly produced the type I cytokine IFN-γ and the type 2 cytokine IL-5 at on average similar concentrations, suggesting the presence of both Th1 and Th2 cells in these tumors **(**Fig. 4**)**. Only low concentrations of TNF-α, IL-4 and IL-10 were found.

**Fig. 5:**
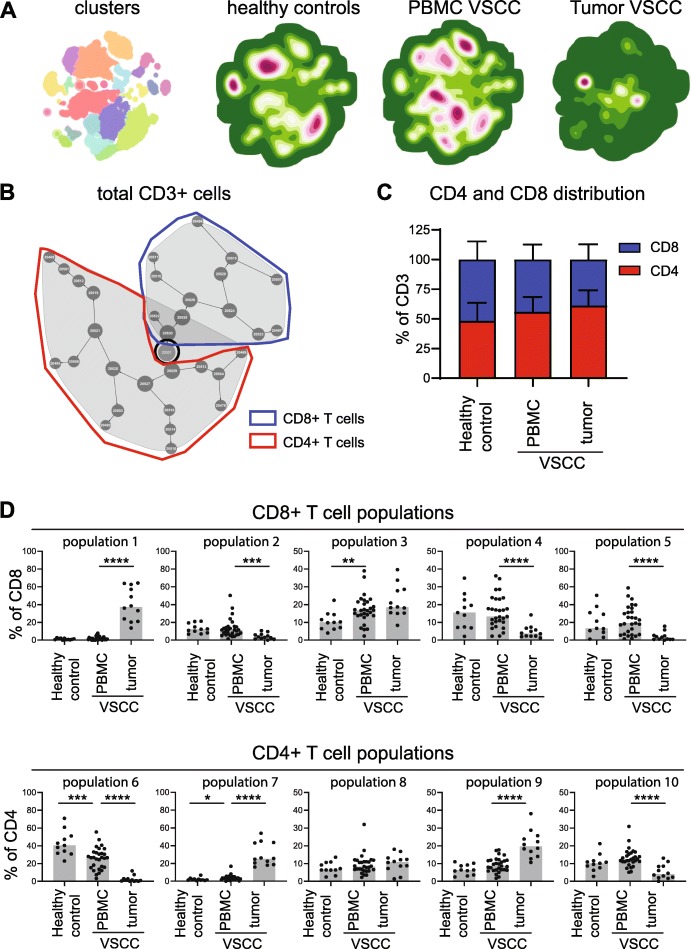
HPVnegVSCC are infiltrated with highly activated CD4^+^ and CD8^+^ effector/memory T cells. PBMC of healthy controls (*n* = 11) as well as PBMC (*n* = 29) and freshly dissociated tumor-derived TIL (*n* = 12) of HPVnegVSCC patients were analyzed by 13-parameter flow cytometry analysis. **a** Hierarchical Stochastical Neighbor Embedding (HSNE) clusters (left) and density plots (right) visualizing the high-dimensional flow cytometry data in two dimensions for the collective total CD3^+^ T cells for indicated groups. The identified cell subsets are identified in the cluster plots by the different colors. **b** CITRUS automatic discovery of stratifying biological signatures within tumor and blood samples visualizes 10 distinctive populations of CD8^+^ and CD4^+^ T cells the total CD3^+^ immune population. Every cell population represented by a node is divided on basis of median level of expression of a differently expressed marker into two new nodes (cellular subsets) going from the center (all cells) to the periphery of the plot. **c** The distribution of CD4^+^ and CD8^+^ T-cell frequencies (mean ± SEM) within the total CD3^+^ T cell population is depicted for healthy control and VSCC PBMC and tumors. **d** Scatter plots with bars displaying frequencies of CD8^+^ (# 1 to 5; top panel) and CD4^+^ (# 6 to 10; bottom panel) T cell populations are given as % of CD8^+^ and CD4^+^ cells. (*p < 0.05, **p < 0.01, ***p < 0.001 and ****p < 0.0001)

To analyze the tumor-infiltrating T cells an antibody mix against CD45, CD3, CD4, CD8, CCR7, CD45RA, CD103, CD161, PD-1, CD38, HLA-DR and NKG2A was used to stain the fresh VSCC digests. In addition, PBMC of healthy female controls (*n* = 11) and PBMC of HPVnegVSCC (*n* = 29) were stained. A combined hierarchical Stochastical Neighbor Embedding (HSNE) analysis of the high-dimensional single cell data obtained from staining the blood and tumor samples resulted in the identification of several distinct immune populations (clusters), which were more prominently present or absent in the tumors or PBMC of the VSCC patients **(**Fig. 5a**)**. In order to automatically discover stratifying biological signatures within VSCC blood and tumor samples, we made use of the automated and data-driven CITRUS platform, as an unbiased and thorough correlation-based tool for mining and inspection of cell subsets at the single cell level nested within high-dimensional datasets [34]. The CITRUS analysis resulted in ten distinctive (groups of) lymphocyte populations significantly higher present in one or more of the three different types of samples **(**Fig. 5b**)**. The CD4 and CD8 distribution within the total CD3^+^ T-cell population did not differ between PBMC and tumors of VSCC patients **(**Fig. 5c**).** Of the five identified CD8^+^ T-cell subsets, populations 2, 4, and 5 were significantly underrepresented in VSCC tumors **(**Fig. 5d**)**. Population 2 comprised CD8^+^CD161^+^PD1^+^CD38^+^ HLA-DR^−^ effector memory RA^+^ T cells (Temra), whereas population 4 (CD8^+^ Tcm/em) and population 5 (CD8^+^ Temra) did not display these markers **(**Additional file 11**)**. The CD8^+^ T-cell population (#1) which was clearly overrepresented in VSCC tumors, consisted of CD8^+^CD103^+^CD161^−^NKG2A^+/−^PD1^++^CD38^++^HLA-DR^+^ Tem cells. Of the five different CD4^+^ T-cell subsets identified, population 6 (CD4^+^PD-1^−^CD161^−^CD38^++^HLA-DR^−^ naïve T cells) and population 10 (CD4^+^PD-1^+^CD38^−^CD161^−^HLA-DR^−^ Tem/cm) were lower in VSCC than PBMC. In contrast, two populations of CD4^+^ effector T cells were found at significantly higher percentages and comprised CD4^+^PD-1^++^CD161^−^CD38^+^HLA-DR^+^Tem (#7) as well as CD4^+^PD-1^−^CD161^−^CD38^−^HLA-DR^−^Tcm/em (#9). The co-expression of PD-1, CD38 and HLA-DR is indicative for T-cell activation. As such the strong tumor-specific infiltration of HPVnegVSCC with activated CD8^+^ (population 1) and CD4^+^ (population 7) effector T cells sustains the notion these tumors are immunogenic and explain why their presence is associated with better clinical outcome.

**Fig. 6:**
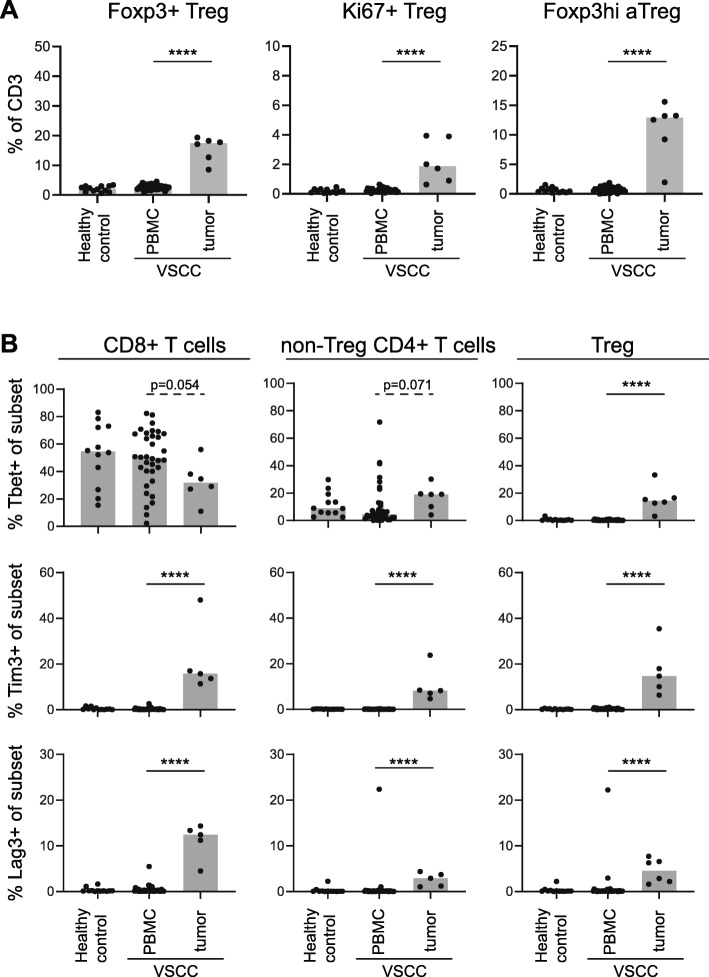
HPVnegVSCC are infiltrated with activated and Tbet-expressing CD4^+^ and CD8^+^ T cells and Tregs. PBMC of healthy controls (*n* = 12) and PBMC (*n* = 34) and freshly dispersed tumors (*n* = 6) of HPVnegVSCC patients were analyzed by 13-parameter flow cytometry analysis. Scatter plots with bars displaying **(a)** frequencies of total Foxp3^+^ Tregs (left) and proliferating (Ki67^+^; middle) and Foxp3hi activated Tregs (Foxp3^hi^ aTregs; right) within CD3^+^ T cells and **(b)** frequencies of Tbet^+^ (top), Tim-3^+^ (middle) and Lag-3^+^ (bottom) cells within the CD8^+^ (left), non-Treg CD4^+^ (middle) and Foxp3^+^ Treg (right) T cell populations. (*p < 0.05, **p < 0.01, ***p < 0.001 and ****p < 0.0001)

For half (6/12) of the freshly digested VSCC samples enough material was available to characterize the T cell infiltrate with a second antibody mix against CD3, CD4, CD8, CD25, CD127, Foxp3, Tim-3, Lag-3 and Tbet. These samples were analyzed for the presence of different types of Tregs, Tbet+ cells and the two checkpoint molecules according to the strategy shown in Additional file 12. Similar to what was found in the FFPE tissue samples, a tumor-specific increase in activated and proliferating (Ki67^+^) Tregs was observed **(**Fig. 6a**)**. Furthermore, tumor-specific increases in the percentages of Tim-3 and Lag-3 Tregs, CD8^+^ and non-Treg CD4^+^ T cells were observed **(**Fig. 6b**)**, confirming that part of the tumor-infiltrating T cells has been activated. Last but not least, on average 30% of the CD8^+^ and 20% of the non-Treg CD4^+^ T cells expressed the transcription factor Tbet, which is in line with the IFN-γ production of the cultured TILs. Finally, only a small percentage of the Tregs expressed Tbet **(**Fig. 6b**)**.

Based on the cytokine production and the expression of several checkpoints, transcription factors and activation markers, we conclude that HPVnegVSCC are infiltrated with variable numbers of activated type 1 and 2 CD8^+^ and CD4^+^ effector T cells as well as Tregs.

## Discussion

We asked the question if the observed differences in recurrence rate and survival between the three subtypes of VSCC [14, 17], classified by the presence of HPV, the overexpression of p53 or the absence of both, may have an immunological background. Our study is the first to show that a strong infiltration of the tumor cell nests with helper (CD3^+^CD8^−^Foxp3^−^) T cells is important for clinical outcome after primary surgery, irrespective of whether the VSCC are caused by HPV or other oncogenic pathways, including *TP53* mutations. Probable reasons for the failure to detect this association in previous studies [6, 9–11] are related to the importance of the location of the T cells in the tumor and the homogeneity in stage and treatment of the VSCC patients analyzed. In line with the RFP and the percentage of recurrences found in each of the three subtypes in VSCC, the percentage of tumors with high intraepithelial helper T-cell infiltration was the highest in the HPV-driven VSCC (78%), followed by VSCC not associated with HPV or p53 overexpression (60%), and the lowest in VSCC with abnormal p53 expression (40%). Importantly, these data suggest that T-cell infiltration of VSCC may be influenced by the oncogenic pathway underlying the development of a particular VSCC.

Although the helper T cells display the strongest relationship with clinical outcome, this does not mean that CD8^+^ T cells are not important in VSCC. Whenever there is a strong intraepithelial infiltration with T cells, this is because both subsets of T cells are present in large numbers. Furthermore, we noted a positive association between the intraepithelial presence of CD3^+^PD-1^+^ T cells and clinical outcome. In-depth flow cytometric analysis revealed that this PD1^+^ T-cell population comprised activated CD4^+^PD-1^++^CD161^−^CD38^+^HLA-DR^+^ and CD8^+^CD103^+^CD161^−^NKG2A^+/−^PD1^++^CD38^++^HLA-DR^+^ effector memory T cells. Potentially, helper T cells play an important role in VSCC because a substantial fraction of VSCC can partially downregulate HLA class I expression whilst the levels of tumor-expressed HLA class II may go up [35]. Based on the percentages of T cells co-expressing the transcription factor Tbet, as counted in the tumor sections and measured in fresh VSCC by flow cytometry, and by the detection of IFN-γ and IL-5 in the supernatants of stimulated TIL, the VSCC-infiltrating T cells are both of a type 1 and 2 phenotype.

Current treatment of VSCC does not take into account the differences in etiology and clinical outcome [3]. The relationship between T-cell infiltration and clinical outcome suggests that immunotherapy may form a new treatment option for VSCC, as in other tumor types this was associated with a better response to immunotherapy [32, 33]. These other cancer types were categorized into immune-inflamed (hot), − altered (excluded or suppressed) and -deserted (cold) tumors in order to define which immunotherapeutic (combination) approach may work best. For instance, hot tumors show the best response to checkpoint blockade (e.g. anti-PD1 and anti-CTLA-4) [32, 33]. Also VSCC could be divided according to these four immune phenotypes. Only a few (5 of 65) VSCC were categorized as truly immune-inflamed while a substantial portion (37%, *n* = 24) displayed the immune altered-excluded phenotype. However, the latter group displayed a significant stronger intraepithelial T cell infiltration when compared to the immune altered-suppressed phenotype and showed a better OS. Patients with inflamed and immune altered-excluded VSCC may be selected for treatment with immunotherapy. In our study high percentages of intratumoral T cells expressed PD-1. Others found variable percentages of cases in which the VSCC (> 30%) or VSCC-infiltrating immune cells (> 90%) expressed PD-L1 [11, 36, 37], congruent with our observation that there are varying numbers of tumor-infiltrating T cells which can produce IFN-γ, as indicated by expression of Tbet, and may lead to adaptive PD-L1 expression [38]. Altogether, this makes a strong case for the treatment of inflamed and immune altered-excluded VSCC with PD-1/PD-L1 checkpoint therapy. Indeed, a first successfully treated case with advanced stage recurrent vulvar cancer has been reported with PD-L1 blockade [39]. A tumor-specific increase of the CD4+ T-cell response seems more likely to be achieved by CTLA4-blockade than by targeting PD-1 [40], arguing for a combination of PD-L1 and CTLA-4 blockade to reinvigorate the tumor-specific CD4+ T-cell response. Another option would be the use of an agonistic antibody to OX-40 [41], which in combination with PD-L1 blockade displayed synergistic effects on CD4+ T-cell reactivity [40]. Moreover, treatment of the generally T-cell infiltrated HPVposVSCC may include therapeutic HPV16 vaccination as HPV-driven oropharyngeal cancers responded well to the combination of checkpoint therapy and therapeutic vaccination [42].

One-third of our VSCC were phenotyped as immune-deserted or cold tumors which may exist because of a lack of antigens or their presentation (immune ignorance), or because of several deficits leading to a lack of priming or to tolerance [32, 42]. Apart from the HPV-induced VSCC, the viral proteins of which may drive a strong T-cell response similar to what is seen in HPV-driven oropharyngeal cancer [24], 40–60% of the HPVnegVSCC display strong intraepithelial T cell infiltration. This suggests that also in these tumors immunogenic tumor antigens are expressed and presented. Currently, the antigens recognized by T cells in HPVnegVSCC are unknown but the majority of primary VSCC express for instance the well-known tumor antigens MAGEA1 and MAGEA4 [43], but it is unknown if these antigens function as target for the VSCC-infiltrating T-cells as this still needs to be studied. We have no data on a direct relation between the level of T-cell infiltration and the availability of strong tumor antigens expressed by VSCC, but our work on HPV-specific immunity in different cancers [25], as well as that of others in for instance melanoma [44–46], suggest that this is not the case. More likely, a lack of inflammation or danger signals has played a role in HPVnegVSCC. Intratumoral activation of the Stimulator of Interferon Genes (STING) pathway [44], the use of oncolytic viruses [45], but also intratumoral injections of toll-like receptor (TLR)-agonists [46] have been shown to sensitize cold tumors to checkpoint blockade. For cold VSCC tumors, the TLR7/8-agonist imiquimod may be a promising topically applied therapeutic agent. Imiquimod elevates numerous genes involved in the regulation of innate immunity, resulting in the migration of DC to the application site, and subsequently the activation of a type 1 T-cell response [47]. Patients with a precancerous lesion of HPVposVSCC responded very well to imiquimod therapy [48]. Notably, imiquimod treatment of breast metastases in the skin not only converted them from cold to hot, as demonstrated by a profound infiltration with CD4+ and CD8+ T cells, but also led to tumor regression [49].

There are several limitations to our study. The correlation between better clinical outcome and intraepithelial T-cell infiltration was found in a highly homogeneous patient group with early stage cancer and treated with surgery. If this relationship also exists in locally advanced cancer patients treated with (chemo) radiotherapy has to be determined. Furthermore, next to the median cell count, we optimized the chance to detect a statistically significant relation between T-cell subsets and clinical outcome in a relatively small group of patients. Hence our results need to be validated in a larger cohort. In addition, our data suggests that the etiology of the VSCC may have an impact on its immunogenicity. While this would fit with the concept that different oncogenic pathways may influence local immunity [18, 19], the numbers of VSCC analyzed are such that the outcome can only be used for hypothesis generation. Moreover, we have not analyzed the myeloid cell component, which on itself may impact prognosis and T-cell function. Finally, less than 20% of VSCC are induced by HPV. As such, we merely collected small pieces of fresh tumor tissue and PBMCs from HPVnegVSCC patients. A more extensive comparison of the tumor-infiltrating immune phenotypes between HPV-induced and HPVnegVSCC, therefore, was not possible.

In conclusion, our observation that a strong coordinated intraepithelial infiltration with T cells is highly associated with a better clinical course of early stage VSCC after surgery and suggests that this group of patients may benefit from immunotherapy as an alternative to potential mutilating surgery in this delicate anatomical area [3]. In parallel to the use of the different categories of immune cell infiltrated tumors in other types of cancer [32, 33], these tumor classifications can also be used to tailor immunotherapy approaches in VSCC. Near future studies should focus on the effects of checkpoint blockade in patients classified with inflamed and altered-excluded tumors while patients diagnosed with an immune-deserted (cold) VSCC may benefit more from therapies that induce acute inflammation. Furthermore, future studies should assess the mutational landscape of VSCC as this will reveal if a tumor-specific (neo)-antigen T-cell repertoire could be harnessed to treat the HPVnegVSCC as well as assess if the HPV-specific T-cell repertoire can be exploited to treat HPVposVSCC.

## Additional files

Additional file 1: Antibody panels. (DOCX 19 kb)

Additional file 2: Patient characteristics of FFPE cohort. (DOCX 23 kb)

Additional file 3: HPVposVSCChave better overall survival compared to HPVnegVSCC. (PDF 260 kb)

Additional file 4: Tissue segmentation and image analysis by VECTRA and total T cell infiltrate in VSCC subtypes and healthy controls. (PDF 419 kb)

Additional file 5: Statistical differences in T-cell infiltration between VSCC subtypes and healthy controls. (DOCX 16 kb)

Additional file 6: Pearson correlations between the numbers of intraepithelial and stromal T cells. (DOCX 14 kb)

Additional file 7: Differences in survival for the four immune categories of VSCC. (PDF 300 kb)

Additional file 8: Median and optimized cut-off point by ROC curve analysis per phenotype and outcome. (DOCX 17 kb)

Additional file 9: Clinical impact of several subsets of intraepithelial T cells in the total group of VSCC and in HPVnegVSCCpatients only. (PDF 399 kb)

Additional file 10: Uni- and multivariate analysis for recurrence-free period. (DOCX 18 kb)

Additional file 11: Clustering analysis using CITRUS revealed 10 distinctive populations of CD4+ and CD8+ T cells. (PDF 643 kb)

Additional file 12: Gating strategy for CD8+, non-Treg CD4+ and regulatory (Treg) T-cell populations. (PDF 263 kb)

## Data Availability

All data generated or analyzed during this study are included in this published article (and its additional files) and are available from the corresponding author on reasonable request.

## Abbreviations

Anti-CTLA-4: Anti-cytotoxic T-lymphocyte-associated protein 4
Anti-PD1: Anti-programmed cell death protein 1
BSA: Bovine serum albumin
CTLA-4: Cytotoxic T-lymphocyte-associated protein 4
FCS: Fetal calf serum
FFPE: Formalin-fixed paraffin embedded
HLA: Human leukocyte antigen
HPV: Human papilloma virus
HPVnegVSCC: Human papilloma virus negative vulvar squamous cell carcinoma
HPVnegVSCC/p53abn: Human papilloma virus negative vulvar squamous cell carcinoma with p53 abnormal expression
HPVnegVSCC/p53wt: Human papilloma virus negative vulvar squamous cell carcinoma with p53 wildtype expression
HPVposVSCC: Human papilloma virus positive vulvar squamous cell carcinoma
IDO: Indoleamine 2,3-dioxygenase
IFNγ: Interferon-gamma
IL-10: Interleukin-10
IL-4: Interleukin-4
IL-5: Interleukin-5
MAGEA1: Melanoma-associated antigen 1
MAGEA4: Melanoma-associated antigen 4
OS: Overall survival
p53wt: p53 wildtype
p53abn: p53 abnormal
PBMC: Peripheral blood mononuclear cells
PBS: Phosphate buffered saline
PD-1: Programmed cell death protein 1
PD-L1: Programmed cell death ligand 1
PHA: Phytohemagglutinin
RFP: Recurrence-free period
ROC: Receiver operating characteristics
STING: Stimulator of interferon genes
Tcm: Central memory T cell
Tem: Effector memory T cell
Temra: Effector memory RA+ T cells
Th1: T-helper 1
Th2: T-helper 2
TIL: Tumor infiltrating lymphocyte
TNF-α: Tumor necrosis factor alpha
Treg: Regulatory T cell
VSCC: Vulvar squamous cell carcinoma
