# Supplementary material for: High numbers of activated helper T cells are associated with better clinical outcome in early stage vulvar cancer, irrespective of HPV or p53 status
Source: J Immunother Cancer. 2019 Sep 3;7:236. doi: 10.1186/s40425-019-0712-z (PMC6724316; doi:10.1186/s40425-019-0712-z)
Supplement: Supplementary file 1 — Antibody panels. (DOCX 19 kb) [file 40425_2019_712_MOESM1_ESM.docx]

|  | **Antibody** | **Antigen** | **Detection** | **Fluorochrome** | **Clone** | **Supplier** |
| --- | --- | --- | --- | --- | --- | --- |
| **Multiplex immunofluroescent staining** | panel 1 |  |  |  |  |  |
|  | 1 | Pan-cytokeratin | Direct | Alexa647 | AE1/AE3, C11 | Thermo fisher scientific |
|  | 2 | PD1 | Indirect | OPAL520 | D4W2J | Cell signaling Technology |
|  | 3 | CD8 | Indirect | CF555 | 4B11 | DAKO |
|  | 4 | Foxp3 | Indirect | CF633 | 236A/E7 | eBioscience |
|  | 5 | CD3 | Direct | Alexa594 | D7A6E | Cell signaling Technology |
|  |  | DAPI |  |  |  |  |
|  | panel 2 |  |  |  |  |  |
|  | 1 | Pan-cytokeratin | Direct | Alexa647 | AE1/AE3, C11 | Thermo fisher scientific |
|  | 2 | Tbet | Indirect | OPAL520 | H-210 | Santa Cruz |
|  | 3 | CD3 | Direct | Alexa594 | D7A6E | Cell signaling Technology |
|  |  | DAPI |  |  |  |  |
|  |  |  |  |  |  |  |
| **Flowcytometry antibody panel** | 1 | CD45 |  | PerCP-Cy5.5 | 2D1 | BD Biosciences |
|  | 2 | CD3 |  | V450 | UCHT1 | BD Biosciences |
|  | 3 | CD4 |  | AlexaFluor700 | RPA-T4 | BD Biosciences |
|  | 4 | CD8 |  | PE-CF594 | RPA-T8 | BD Biosciences |
|  | 5 | CD103 |  | BV605 | Ber-ACT8 | Biolegend |
|  | 6 | CD161 |  | PE | HP-3G10 | Biolegend |
|  | 7 | CD38 |  | BV650 | HB-7 | Biolegend |
|  | 8 | HLA-DR |  | V500 | G46-6 | BD Biosciences |
|  | 9 | PD-1 |  | PE-Cy7 | EH12.2H7 | Biolegend |
|  | 10 | NKG2a |  | APC | Z199 | Beckman Coulter |
|  | 11 | CD45RA |  | APC-H7 | HI100 | BD Biosciences |
|  | 12 | CCR7 |  | AlexaFluor488 | G043H7 | Biolegend |
|  | 13 | CD3 |  | V500 | UCHT1 | BD Biosciences |
|  | 14 | CD8 |  | BB700 | HIT8a | BD Biosciences |
|  | 15 | CD25 |  | PE-Cy7 | 2A3 | BD Biosciences |
|  | 16 | CD127 |  | BV650 | A019D5 | Biolegend |
|  | 17 | TIM-3 |  | BV605 | F38-2E2 | Biolegend |
|  | 18 | LAG-3 |  | BV421 | [11C3C65](https://www.biolegend.com/en-us/search-results?Clone=11C3C65) | Biolegend |
|  | 19 | KLRG-1 |  | APC | 13F12F2 | eBiosciences |
|  | 20 | Foxp3 |  | PE-CF594 | 259D/C7 | BD Biosciences |
|  | 21 | Ki67 |  | FITC | 20Raj1 | eBiosciences |
|  | 22 | Tbet |  | PE | ebio4B10 | eBiosciences |
|  |  |  |  |  |  |  |

**Additional file 1. Antibody panels**
