# Supplementary material for: High numbers of activated helper T cells are associated with better clinical outcome in early stage vulvar cancer, irrespective of HPV or p53 status
Source: J Immunother Cancer. 2019 Sep 3;7:236. doi: 10.1186/s40425-019-0712-z (PMC6724316; doi:10.1186/s40425-019-0712-z)
Supplement: Supplementary file 2 — Patient characteristics of FFPE cohort. (DOCX 23 kb) [file 40425_2019_712_MOESM2_ESM.docx]

| Characteristic (n, %) | HPVpos  (n=23) | HPVneg/p53wt  (n=20) | HPVneg/p53abn  (n=22) | *p*-value |
| --- | --- | --- | --- | --- |
| Age – yr (median, range) | 60 (40-89) | 70.5 (47-91) | 77.5 (41-86) | **0.013** |
| Smoking (n, %) |  |  |  | **0.005** |
| No | 5 (21.7%) | 12 (60%) | 14 (63.6%) |  |
| Current | 15 (65.2%) | 3 (15%) | 3 (13.6%) |  |
| Non-current | 1 (4.3%) | 2 (10%) | 1 (4.5%) |  |
| Unknown | 2 (8.7%) | 3 (15%) | 4 (18.2%) |  |
| Immunosuppression |  |  |  | - |
| HIV | - | - | - |  |
| Immunosuppressive | 2 (8.7%) | - | - |  |
| FIGO stadium |  |  |  | 0.429 |
| IA | 2 (8.7%) | 1 (5%) | - |  |
| IB | 21 (91.3%) | 19 (95%) | 21 (95.5%) |  |
| II | - | - | - |  |
| IIIA | - | - | 1 (4.5%) |  |
| Size tumor in cm |  |  |  | 0.450 |
| ≤2cm | 15 (65.2%) | 9 (45%) | 10 (45.5%) |  |
| 2-4cm | 5 (21.7%) | 7 (35%) | 6 (27.3%) |  |
| ≥4cm | 2 (8.7%) | 4 (20%) | 6 (27.3%) |  |
| Depth of invasion in mm |  |  |  | 0.129 |
| ≤4mm | 15 (65.2%) | 7 (35%) | 11 (50%) |  |
| >4mm | 7 (30.4%) | 12 (60%) | 11 (50%) |  |
| LVSI |  |  |  | 0.414 |
| No | 20 (87%) | 19 (95%) | 18 (81.8%) |  |
| Yes | 1 (4.3%) | - | 3 (13.6%) |  |
| Unknown | 2 (8.7%) | 1 (5%) | 1 (4.5%) |  |
| Perineural growth |  |  |  | 0.245 |
| No | 10 (43.5%) | 12 (60%) | 9 (40.9%) |  |
| Yes | - | - | 2 (9.1%) |  |
| Unknown | 13 (56.5%) | 8 (40%) | 11 (50%) |  |
| Positive margin |  |  |  | 0.371 |
| No | 23 (100%) | 20 (100%) | 21 (95.5) |  |
| Yes | - | - | 1 (4.5%) |  |
| HPV genotyping |  |  |  | - |
| Type 16 | 19 (82.6%) | - | - |  |
| Type 18 | 1 (4.3%) | - | - |  |
| Type 33 | 2 (8.6%) | - | - |  |
| Type 53 | 1 (4.3%) | - | - |  |
| Treatment modality |  |  |  | 0.646 |
| Surgery | 22 (95.7%) | 19 (95%) | 21 (95.5%) |  |
| Surgery & (chemo)radiotherapy | 1 (4.3%) | 1 (5%) | 1 (4.5%) |  |
| Recurrence |  |  |  | **0.006** |
| No | 20 (87%) | 12 (60%) | 9 (40.9%) |  |
| Yes | 3 (13%) | 8 (40%) | 13 (59.1%) |  |
| Local (first recurrence) | 3 (100%) | 6 (75%) | 12 (92.3%) |  |
| Locoregional (first recurrence) | - | 2 (25%) | 1 (7.7%) |  |
| Distant (first recurrence) | - | - | - |  |
| Time to first recurrence in months (median, range) | 30 (9-42) | 34 (11-103) | 32 (7-125) | 0.794 |
| Survival status |  |  |  | **0.014** |
| Alive | 22 (95.7%) | 14 (70%) | 13 (59.1%) |  |
| Dead | 1 (4.3%) | 6 (30%) | 9 (40.9%) |  |

**Additional file 2. Patient characteristics of FFPE cohort**

LVSI = lymphovascular space invasion. Significant *p*-values <0.05 by group comparison analysis of categorical data by chi-square test are shown in bold. For non-parametric continuous variables the Mann-Whitney U test was used.
