# Supplementary material for: High numbers of activated helper T cells are associated with better clinical outcome in early stage vulvar cancer, irrespective of HPV or p53 status
Source: J Immunother Cancer. 2019 Sep 3;7:236. doi: 10.1186/s40425-019-0712-z (PMC6724316; doi:10.1186/s40425-019-0712-z)
Supplement: Supplementary file 3 — HPVposVSCChave better overall survival compared to HPVnegVSCC. (PDF 260 kb) [file 40425_2019_712_MOESM3_ESM.pdf]

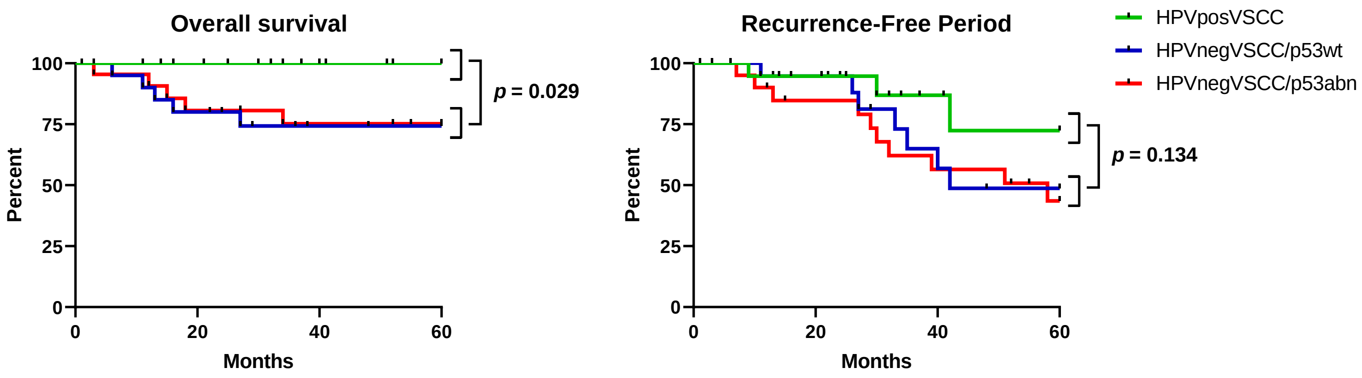

Additional file 3. HPVposVSCC have better overall survival compared to HPVnegVSCC. Kaplan-Meier curves showing overall survival (left) and the recurrence free period (RFP; right) for VSCC patients with HPVposVSCC (green), HPVnegVSCC/p53wt (blue) and HPVnegVSCC/p53abn (red). Statistical significance of the survival distribution was analyzed by log-rank testing. Significant differences between the overall survival of HPVposVSCC and HPVnegVSCC/p53wt (HR=0.14 (95% CI 0.02-0.80), *p*=0.03) or HPVnegVSCC/p53abn (HR=0.15 (95% CI 0.03-0.87), *p*=0.04) is observed. Comparison of the RFP showed similar differences between the groups. HPVposVSCC have better clinical outcome than HPVnegVSCC/p53wt (HR=0.48 (95% CI 0.14-1.60), *p*=0.25) and HPVnegVSCC/p53abn (HR=0.41 (95% CI 0.14-1.24), *p*=0.11).
