# Supplementary material for: High numbers of activated helper T cells are associated with better clinical outcome in early stage vulvar cancer, irrespective of HPV or p53 status
Source: J Immunother Cancer. 2019 Sep 3;7:236. doi: 10.1186/s40425-019-0712-z (PMC6724316; doi:10.1186/s40425-019-0712-z)
Supplement: Supplementary file 4 — Tissue segmentation and image analysis by VECTRA and total T cell infiltrate in VSCC subtypes and healthy controls. (PDF 419 kb) [file 40425_2019_712_MOESM4_ESM.pdf]

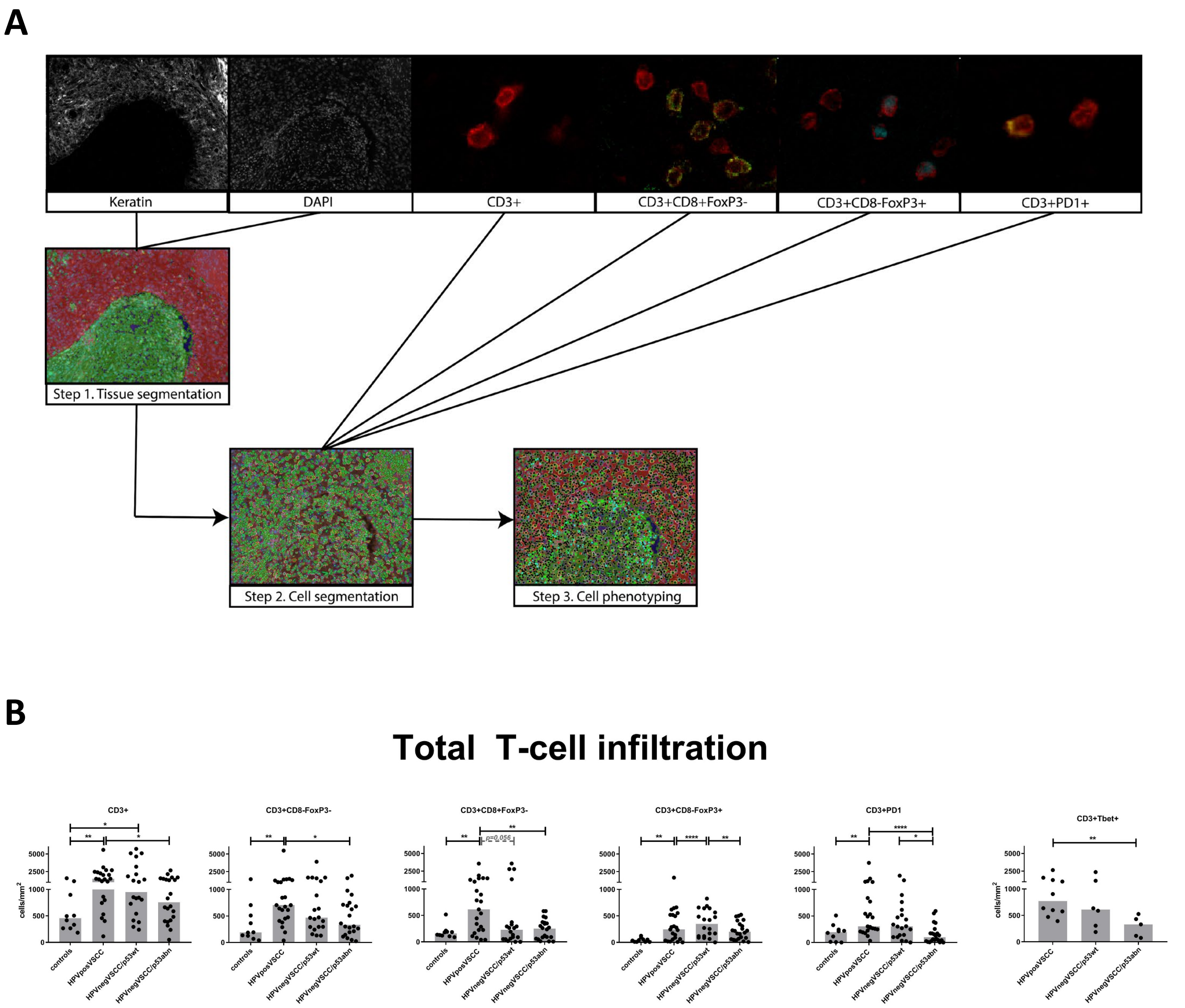

Additional file 4. Tissue segmentation and image analysis by VECTRA and total T cell infiltrate in VSCC subtypes and healthy controls. (**a**) Imaging analysis and spectral separation of dyes was performed with the InForm Cell Analysis software (Perkin Elmer) by using spectral libraries defined with single-marker immunofluorescence detection. All images were segmented into tumor, stroma, and 'no tissue' areas with manual training based on keratin and DAPI. Subsequently, cellular segmentation was performed using counterstain-based approach with DAPI to segment membrane markers (CD3, CD8, and PD1) and nuclei markers (Foxp3). The notorious autofluorescence of erythrocytes was excluded by the absence of DAPI. The phenotype training ($CD3^+$ total, $CD3^+CD8^-Foxp3^-$, $CD3^+CD8^+Foxp3^-$, $CD3^+CD8^-Foxp3^+$) was done semi-automatically after manual training per tumor where at least 20 cells per phenotype were defined. $CD3^+PD1^+$ phenotype was analyzed in a separate training session.

A group of 65 archived tissues sections from VSSC patients and 10 labia from healthy women were simultaneously analyzed for the expression of CD3, CD8, Foxp3, PD-1, and pan-keratin as described above. The total number of intraepithelial and stroma infiltrating CD3 (T cells), $CD3^+CD8^-Foxp3^-$ (helper T cells), $CD3^+CD8^+Foxp3^-$ (cytotoxic T cells), $CD3^+CD8^-Foxp3^+$ (regulatory T cells) and $CD3^+PD1^+$ T cells are given as cells/mm^2 for HPV-negative healthy labia (n=10), and HPVposVSCC, HPVnegVSCC/p53wt and HPVnegVSCC/p53abn patients (n=23, n=20 and n=22 respectively (**b**). A subcohort was analyzed for the $CD3^+Tbet^+$ T cells in 10 HPVposVSCC, 6 HPVnegVSCC/p53wt and 5 HPVnegVSCC/p53abn. The bars indicate the median cell count, individual samples are indicated by closed circles. Differences between two groups were calculated with a Mann-Whitney test with the significance indicated with asterisks. (*p<0.05, **p<0.01, ***p<0.001 and ****p<0.0001).
