# Supplementary material for: High numbers of activated helper T cells are associated with better clinical outcome in early stage vulvar cancer, irrespective of HPV or p53 status
Source: J Immunother Cancer. 2019 Sep 3;7:236. doi: 10.1186/s40425-019-0712-z (PMC6724316; doi:10.1186/s40425-019-0712-z)
Supplement: Supplementary file 5 — Statistical differences in T-cell infiltration between VSCC subtypes and healthy controls. (DOCX 16 kb) [file 40425_2019_712_MOESM5_ESM.docx]

**Additional file 5. Statistical differences in T-cell infiltration between VSCC subtypes and healthy controls.**

|  | **Controls** | **HPVposVSCC** | **HPVnegVSCC/p53wt** | **HPVnegVSCC/p53abn** | ***p*-value** | ***p*-value** | ***p*-value** | ***p*-value** | ***p*-value** | ***p*-value** |
| --- | --- | --- | --- | --- | --- | --- | --- | --- | --- | --- |
|  | median  (range) | median  (range) | median  (range) | median  (range) | Controls *vs*  HPVposVSCC | Controls *vs*  HPVnegVSCC/p53wt | Controls *vs*  HPVnegVSCC/p53abn | HPVposVSCC *vs*  HPVnegVSCC/p53wt | HPVposVSCC *vs*  HPVnegVSCC/p53abn | HPVnegVSCC/p53wt *vs* HPVnegVSCC/p53abn |
|  | *n*=10 | *n*=23 | *n*=20 | *n*=22 |  |  |  |  |  |  |
| **CD3+ total (E)** | 182,97  (20,76-851,13) | 284,6  (17,0-1383,07) | 242,62  (13,78-2719,33) | 131,83 (12,55-641,09) | 0,144 | 0,397 | 0,675 | 0,697 | **0,029** | 0,059 |
| **CD3+ total (S)** | 241,13 (90,07-872,49) | 1307,51 (33,79-8452,21) | 749,11 (203,67-4719,74) | 618,32 (35,12-2043,65) | **0,002** | 0,004 | 0,018 | 0,368 | **0,018** | 0,290 |
| **CD3+ total (T)** | 456,76 (183,86-1568,55) | 1484,48 (115,01-9011,90) | 949,54 (238,87-5729,06) | 755,90 (49,97-2684,73) | **0,003** | **0,028** | 0,119 | 0,374 | **0,017** | 0,217 |
| **CD3+CD8-Foxp3- (E)** | 68,06 (13,12-774,77) | 124,21 (3,82-606,15) | 72,76 (13,78-2325,93) | 37,70 (2,26-451,38) | 0,269 | 0,713 | 0,483 | 0,609 | **0,026** | 0,102 |
| **CD3+CD8-Foxp3- (S)** | 134,97 (28,79-667,79) | 517,29 (6,47-5138,58) | 401,25 (94,64-1589,67) | 295,60 (18,38-1493,11) | **0,005** | **0,010** | 0,070 | 0,436 | 0,056 | 0,247 |
| **CD3+CD8-Foxp3- (T)** | 190,18 (41,92-1442,56) | 705,26 (37,58-5487,34) | 469,08 (129,82-3915,60) | 332,03 (24,79-1944,50) | **0,010** | 0,044 | 0,268 | 0,336 | **0,028** | 0,257 |
| **CD3+CD8+Foxp3- (E)** | 66,27 (4,42-185,51) | 96,2 (3,57-759,69) | 63,76 (0,00-1222,17) | 41,30 (0,00-169,71) | 0,305 | 0,948 | 0,305 | 0,318 | **0,014** | 0,371 |
| **CD3+CD8+Foxp3- (S)** | 86,90 (20,02-331,15) | 472,48 (20,17-3150,50) | 114,24 (0,00-2506,14) | 119,81 (7,07-483,01) | **0,009** | 0,746 | 0,388 | 0,058 | **0,007** | 0,762 |
| **CD3+CD8+Foxp3- (T)** | 158,18 (88,81-516,66) | 612,94 (40,34-3578,03) | 226,92 (0,00-3580,08) | 249,98 (7,07-581,70) | **0,008** | 0,746 | 0,826 | 0,056 | **0,004** | 0,960 |
| **CD3+CD8-Foxp3+ (E)** | 5,15 (1,72-28,84) | 44,05 (0,00-1383,07) | 80,13 (0,00-285,71) | 26,04 (0,00-268,88) | **0,006** | 0,0002 | **0,012** | **0,008** | 0,725 | **0,026** |
| **CD3+CD8-Foxp3+ (S)** | 27,11 (1,85-96,61) | 173,04 (0,00-1631,58) | 193,24 (0,00-793,29) | 152,49 (3,87-458,11) | **0,007** | 0,001 | **0,001** | 0,724 | 0,570 | 0,208 |
| **CD3+CD8-Foxp3+ (T)** | 38,46 (7,96-125,45) | 244,68 (0,00-1642,67) | 347,76 (0,00-1012,27) | 200,43 (6,60-521,08) | **0,004** | 0,0001 | **0,001** | 0,408 | 0,467 | 0,078 |
| **CD3+PD1+ (E)** | 41,94 (0,00-200,55) | 126,32 (5,13-1034,34) | 94,4 (0,00-551,40) | 24,07 (0,00-257,71) | **0,031** | 0,231 | 0,458 | 0,173 | **0,0001** | **0,006** |
| **CD3+PD1+ (S)** | 125,58 (0,00-1041,00) | 281,9 (27,11-3031,92) | 177,69 (0,00-1386,38) | 62,76 (0,00-451,26) | 0,089 | 0,267 | 0,483 | 0,527 | **0,001** | **0,012** |
| **CD3+PD1+ (T)** | 196,16 (0,00-509,04) | 303,85 (32,23-3750,06) | 304,3 (0,00-1937,78) | 99,98 (0,00-593,37) | **0,006** | 0,074 | 0,562 | 0,422 | **0,0001** | **0,012** |

(E)= epithelium, (S) = stroma, (T) = total. *p-*values between two groups were analyzed by non-parametric Mann-Whitney U test to determine differences in T cell subset infiltration between healthy controls, HPVposVSCC, HPVnegVSCC/p53wt, and HPVnegVSCC/p53abn. Significant difference are indicated in bold.
