# Supplementary material for: High numbers of activated helper T cells are associated with better clinical outcome in early stage vulvar cancer, irrespective of HPV or p53 status
Source: J Immunother Cancer. 2019 Sep 3;7:236. doi: 10.1186/s40425-019-0712-z (PMC6724316; doi:10.1186/s40425-019-0712-z)
Supplement: Supplementary file 6 — Pearson correlations between the numbers of intraepithelial and stromal T cells. (DOCX 14 kb) [file 40425_2019_712_MOESM6_ESM.docx]

**Additional file 6. Pearson correlations between the numbers of intraepithelial and stromal T cells.**

|  |  | Intraepithelial cell count | | | | |
| --- | --- | --- | --- | --- | --- | --- |
|  |  | CD3+ | CD3+CD8-Foxp3- | CD3+CD8+Foxp3- | CD3+CD8-Foxp3+ | CD3+PD1+ |
| Intraepithelial cell count | CD3+ |  | 0.870 ***p*=0.000** | 0.766 ***p*=0.000** | 0.551 ***p*=0.000** | 0.462 ***p*=0.000** |
|  | CD3+CD8-Foxp3- |  |  | 0.373 ***p*=0.002** | 0.354 ***p*=0.004** | 0.246 ***p*=0.048** |
|  | CD3+CD8+Foxp3- |  |  |  | 0.378 ***p*=0.002** | 0.590 ***p*=0.000** |
|  | CD3+CD8-Foxp3+ |  |  |  |  | 0.179 *p*=0.153 |
|  | CD3+PD1+ |  |  |  |  |  |
| Invasive border cell count | CD3+ | 0.956 ***p*=0.000** | 0.826 ***p*=0.000** | 0.732 ***p*=0.000** | 0.550 ***p*=0.000** | 0.356 ***p*=0.004** |
|  | CD3+CD8-Foxp3- | 0.858 ***p*=0.000** | 0.978 ***p*=0.000** | 0.367 ***p*=0.003** | 0.383 ***p*=0.002** | 0.174 *p*=0.166 |
|  | CD3+CD8+Foxp3- | 0.742 ***p*=0.000** | 0.370 ***p*=0.002** | 0.953 ***p*=0.000** | 0.379 ***p*=0.002** | 0.487 ***p*=0.000** |
|  | CD3+CD8-Foxp3+ | 0.593 ***p*=0.000** | 0.442 ***p*=0.000** | 0.382 ***p*=0.002** | 0.886 ***p*=0.000** | 0.137 *p*=0.278 |
|  | CD3+PD1+ |  |  |  |  |  |
| Stromal cell count | CD3+ | 0.449 ***p*=0.000** | 0.344 ***p*=0.005** | 0.477 ***p*=0.000** | 0.212 *p*=0.090 | 0.415 ***p*=0.001** |
|  | CD3+CD8-Foxp3- | 0.279 ***p*=0.024** | 0.346 ***p*=0.005** | 0.163 *p*=0.194 | 0.106 *p*=0.399 | 0.338 ***p*=0.006** |
|  | CD3+CD8+Foxp3- | 0.490 ***p*=0.000** | 0.226 ***p*=0.001** | 0.692 ***p*=0.000** | 0.219 *p*=0.079 | 0.348 ***p*=0.004** |
|  | CD3+CD8-Foxp3+ | 0.265 ***p*=0.033** | 0.101 *p*=0.423 | 0.316 ***p*=0.010** | 0.279 ***p*=0.025** | 0.228 *p*=0.068 |
|  | CD3+PD1+ | 0.221 *p*=0.076 | 0.333 *p*=0.797 | 0.389 ***p*=0.001** | 0.120 *p*=0.341 | 0.594 ***p*=0.000** |
