# Supplementary material for: High numbers of activated helper T cells are associated with better clinical outcome in early stage vulvar cancer, irrespective of HPV or p53 status
Source: J Immunother Cancer. 2019 Sep 3;7:236. doi: 10.1186/s40425-019-0712-z (PMC6724316; doi:10.1186/s40425-019-0712-z)
Supplement: Supplementary file 7 — Differences in survival for the four immune categories of VSCC. (PDF 300 kb) [file 40425_2019_712_MOESM7_ESM.pdf]

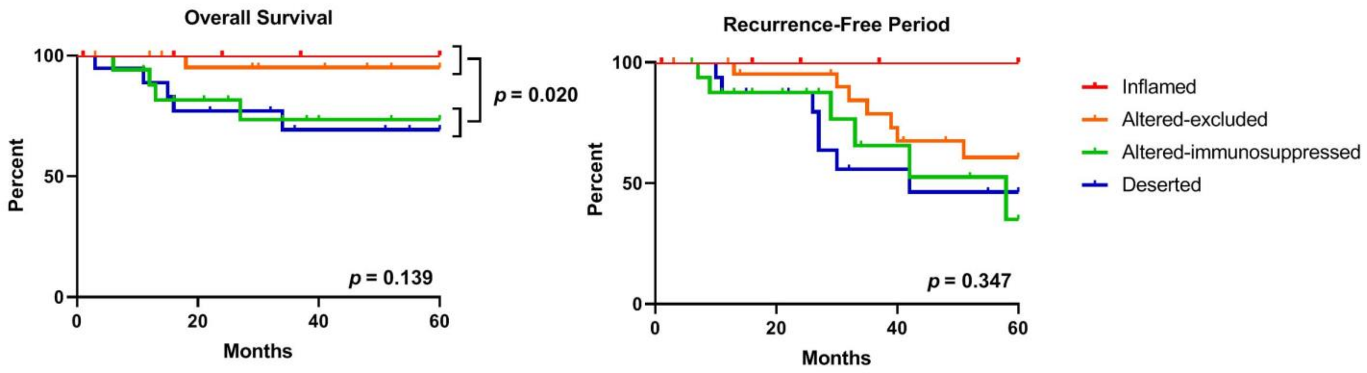

Additional file 7. Differences in survival for the four immune categories of VSCC. Kaplan-Meier curves showing overall survival (left) and the recurrence free period (RFP; right) for VSCC patients with immune-inflamed (red; *n*=5), - altered-excluded (orange; *n*=24), - altered-immunosuppressed (green; *n*=17) and –deserted (blue; *n*=19) T-cell infiltration patterns. Statistical significance of the survival distribution was analyzed by log-rank testing, and differences were considered significant when $p<0.05$. Although the difference between the four groups is not statistically significant, the combined group of immune-inflamed with immune-altered excluded displayed longer overall survival ($p=0.02$) than the combined group of patients with immune-altered suppressed and immune-deserted VSCC.
