# Supplementary material for: High numbers of activated helper T cells are associated with better clinical outcome in early stage vulvar cancer, irrespective of HPV or p53 status
Source: J Immunother Cancer. 2019 Sep 3;7:236. doi: 10.1186/s40425-019-0712-z (PMC6724316; doi:10.1186/s40425-019-0712-z)
Supplement: Supplementary file 8 — Median and optimized cut-off point by ROC curve analysis per phenotype and outcome. (DOCX 17 kb) [file 40425_2019_712_MOESM8_ESM.docx]

**Additional file 8. Median and optimized cut-off point by ROC curve analysis per phenotype and outcome.**

|  | **Recurrence-Free Period** | | | | | |
| --- | --- | --- | --- | --- | --- | --- |
| **Intraepithelial  phenotype** | **ROC  cut-off (cells/mm^2^)** | **HR-ratio  (95% CI)** | ***p*-value** | **Median (cells/mm^2^)** | **HR-ratio  (95% CI)** | ***p*-value** |
| CD3+ | 192.70 | 4.16 (1.59-10.89) | **0.004** | 637.84 | 2.89 (1.17-7.16) | **0.022** |
| CD3+CD8-Foxp3- | 61.82 | 4.85 (1.87-12.59) | **0.001** | 179.1 | 2.84 (1.15-7.01) | **0.024** |
| CD3+CD8+Foxp3- | 37.96 | 1.76 (0.71-4.40) | 0.224 | 115.71 | 1.20 (0.50-2.90) | 0.604 |
| CD3+CD8-Foxp3+ | 38.71 | 1.42 (0.59-3.44) | 0.439 | 77.78 | 1.42 (0.59-3.44) | 0.439 |
| CD3+PD1+ | 99.96 | 2.66 (1.09-6.51) | **0.032** | 130.04 | 2.27 (0.94-5.52) | 0.070 |
|  | **Overall Survival** | | | | | |
|  | **ROC  cut-off (cells/mm^2^)** | **HR-ratio  (95% CI)** | ***p*-value** | **Median (cells/mm^2^)** | **HR-ratio  (95% CI)** | ***p*-value** |
| CD3+ | 309.40 | 4.97 (1.38-17.88) | **0.014** | 637.84 | 8.23 (2.37-28.51) | **0.009** |
| CD3+CD8-Foxp3- | 82.58 | 6.24 (1.79-21.76) | **0.004** | 179.10 | 5.30 )1.53-18.36) | **0.009** |
| CD3+CD8+Foxp3- | 35.63 | 3.00 (0.81-0.09) | 0.097 | 115.71 | 3.21 (0.93-11.09) | 0.065 |
| CD3+CD8-Foxp3+ | 54.58 | 3.01 (0.87-1.42) | 0.083 | 77.78 | 2.48 (0.716-8.59) | 0.152 |
| CD3+PD1+ | 37.67 | 2.95 (0.74-11.69) | 0.125 | 130.04 | 2.62 (0.75-9.14) | 0.132 |

*Significant *p-*values <0.05 are shown in bold.
