# Supplementary material for: High numbers of activated helper T cells are associated with better clinical outcome in early stage vulvar cancer, irrespective of HPV or p53 status
Source: J Immunother Cancer. 2019 Sep 3;7:236. doi: 10.1186/s40425-019-0712-z (PMC6724316; doi:10.1186/s40425-019-0712-z)
Supplement: Supplementary file 9 — Clinical impact of several subsets of intraepithelial T cells in the total group of VSCC and in HPVnegVSCCpatients only. (PDF 399 kb) [file 40425_2019_712_MOESM9_ESM.pdf]

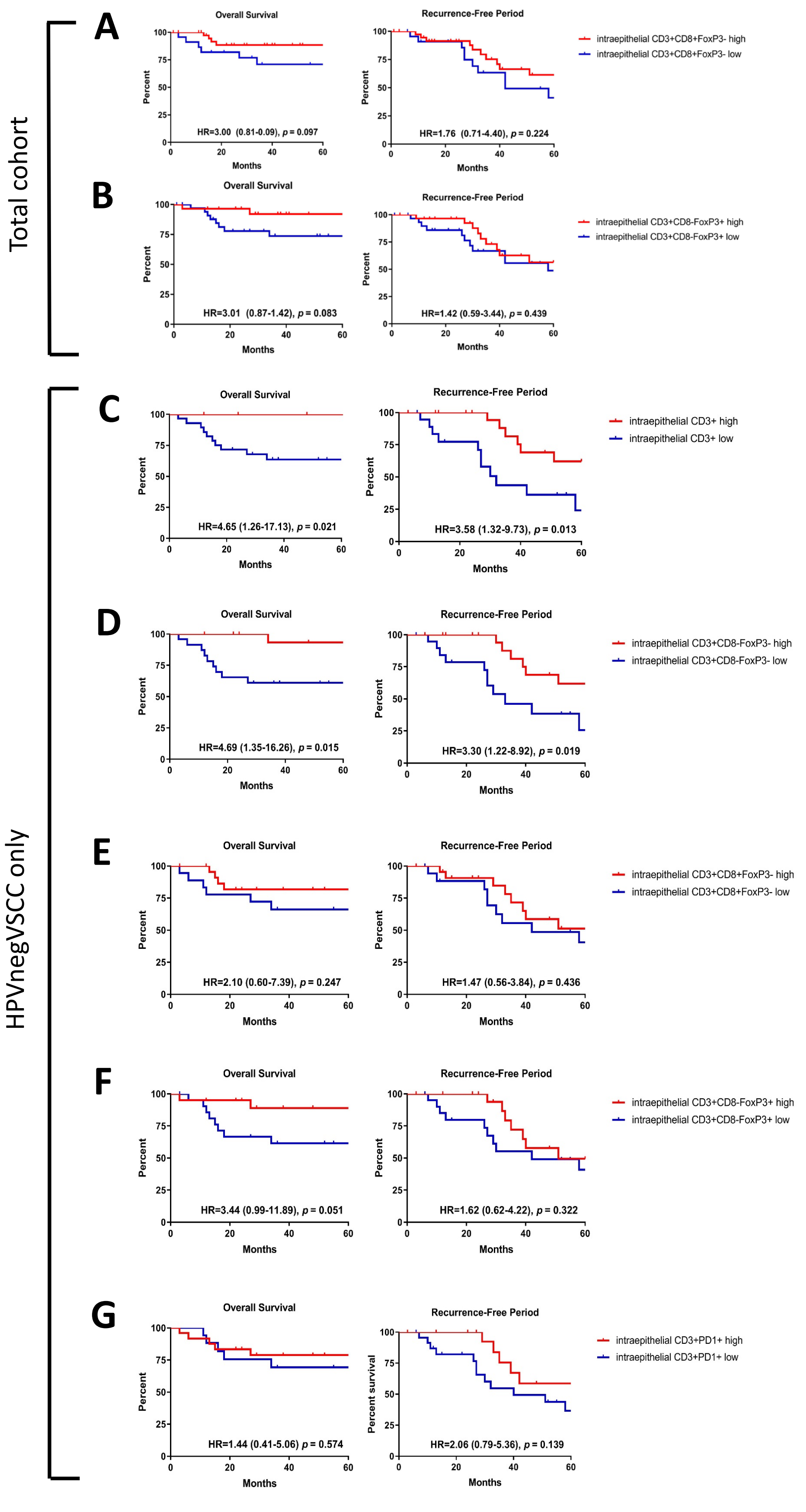

Additional file 9. Clinical impact of several subsets of intraepithelial T cells in the total group of VSCC and in HPVnegVSCC patients only. Kaplan-Meier curves showing overall survival (left) and the recurrence free period (RFP; right) for VSCC patients with high (in red) and low (in blue) numbers of intraepithelial $CD3^{+}CD8^{+}Foxp3^{-}$ (**a**) and intraepithelial $CD3^{+}CD8^{-}Foxp3^{+}$ cells/mm2 (**b**).
Moreover, we calculated the impact of T-cell phenotypes on HPVnegVSCC only (n=42, **c-g**) on clinical outcome. This showed that intraepithelial $CD3^{+}$ (**c**), $CD3^{+}CD8^{-}Foxp3^{-}$ T cells (**d**) retained their positive association with OS and RFP. While other phenotypes in HPVnegVSCC (**e-g**) were not associated with better clinical outcome.

Patients were grouped into the high or low groups based on the best cut-off value for each subset as determined by receiver operating characteristics (ROC) curve analysis. Cut-off values for the total cohort and HPVnegVSCC were similar ($CD3^{+}$ cells were 309.4 cells/mm^2 and 192.7 cells/mm^2 for OS and RFP, respectively. The value of cells/mm^2 were for $CD3^{+}CD8^{+}Foxp3^{-}$ cells 35.63 (OS) and 37.96 (RFP), for $CD3^{+}CD8^{-}Foxp3^{-}$ cells 82.58 (OS) and 61.82 (RFP), for $CD3^{+}CD8^{+}Foxp3^{-}$ cells were 35.63 (OS) and 37.96 (RFP), for $CD3^{+}CD8^{-}Foxp3^{+}$ cells 54.58 (OS) and 38.71 (RFP), and for $CD3^{+}PD1^{+}$ 37.67 (OS) and 99.96 (RPF). Patients with a T cell count below the cut-off value were classified as low, the others as high. A log-rank test was performed to calculate the difference in OS and RFP. Statistical significance of the survival distribution was analyzed by log-rank testing and differences were considered significant when $p<0.05$, as indicated with an asterisk (*$p<0.05$, **$p< 0.01$, ***$p<0.001$ and ****$p<0.0001$).
