# Supplementary material for: High numbers of activated helper T cells are associated with better clinical outcome in early stage vulvar cancer, irrespective of HPV or p53 status
Source: J Immunother Cancer. 2019 Sep 3;7:236. doi: 10.1186/s40425-019-0712-z (PMC6724316; doi:10.1186/s40425-019-0712-z)
Supplement: Supplementary file 10 — Uni- and multivariate analysis for recurrence-free period. (DOCX 18 kb) [file 40425_2019_712_MOESM10_ESM.docx]

|  | Recurrence-Free Period total cohort | | | | Recurrence-Free Period HPVnegVSCC only | | | |
| --- | --- | --- | --- | --- | --- | --- | --- | --- |
| Variable | **HR crude** | ***p*-value** | **HR adjusted** | ***p*-value** | **HR crude** | **p-value** | **HR adjusted** |  |
| Intraepithelial CD3+CD8-Foxp3-  (low/high) | 4.11 (1.62-10.39) | **0.003** | 3.31 (1.22-8.94) | **0.018** | 3.14 (1.15-8.57) | **0.026** | 3.07 (1.07-8.83) | **0.038** |
| Age | 1.04 (1.00-1.07) | **0.037** | 1.02 (0.98-1.06) | 0.281 | 1.02 (0.98-1.06) | 0.321 | 1.01 (0.97-1.05) | 0.586 |
| P53 IHC  (wildtype/abnormal) | 0.62 (0.26-1.49) | 0.283 | 1.33 (0.47-3.72) | 0.591 | 0.85 (0.32-2.23) | 0.734 | 1.21 (0.43-3.43) | 0.721 |
| HPV  (negative/positive) | 2.47 (0.72-8.44) | 0.149 | 1.99 (0.51-7.74) | 0.320 |  |  |  |  |
| Intraepithelial CD3+-  (low/high) | 3.49 (1.41-8.61) | **0.007** | 2.95 (1.05-8.25) | **0.040** | 3.33 (1.22-9.08) | **0.019** | 3.66 (1.19-11.26) | **0.024** |
| Age | 1.04 (1.00-1.07) | **0.037** | 1.02 (0.98-1.06) | 0.288 | 1.02 (0.98-1.06) | 0.321 | 1.01 (0.97-1.05) | 0.688 |
| P53 IHC  (wildtype/abnormal) | 0.62 (0.26-1.49) | 0.283 | 1.53 (0.53-4.43) | 0.438 | 0.85 (0.32-2.23) | 0.734 | 1.50 (0.50-4.45) | 0.470 |
| HPV  (negative/positive) | 2.47 (0.72-8.44) | 0.149 | 2.18 (0.56-8.49) | 0.261 |  |  |  |  |

**Additional file 10. Uni- and multivariate analysis for recurrence-free period.**

*Significant *p-*values <0.05 are shown in bold.
