# Supplementary material for: High numbers of activated helper T cells are associated with better clinical outcome in early stage vulvar cancer, irrespective of HPV or p53 status
Source: J Immunother Cancer. 2019 Sep 3;7:236. doi: 10.1186/s40425-019-0712-z (PMC6724316; doi:10.1186/s40425-019-0712-z)
Supplement: Supplementary file 11 — Clustering analysis using CITRUS revealed 10 distinctive populations of CD4+ and CD8+ T cells. (PDF 643 kb) [file 40425_2019_712_MOESM11_ESM.pdf]

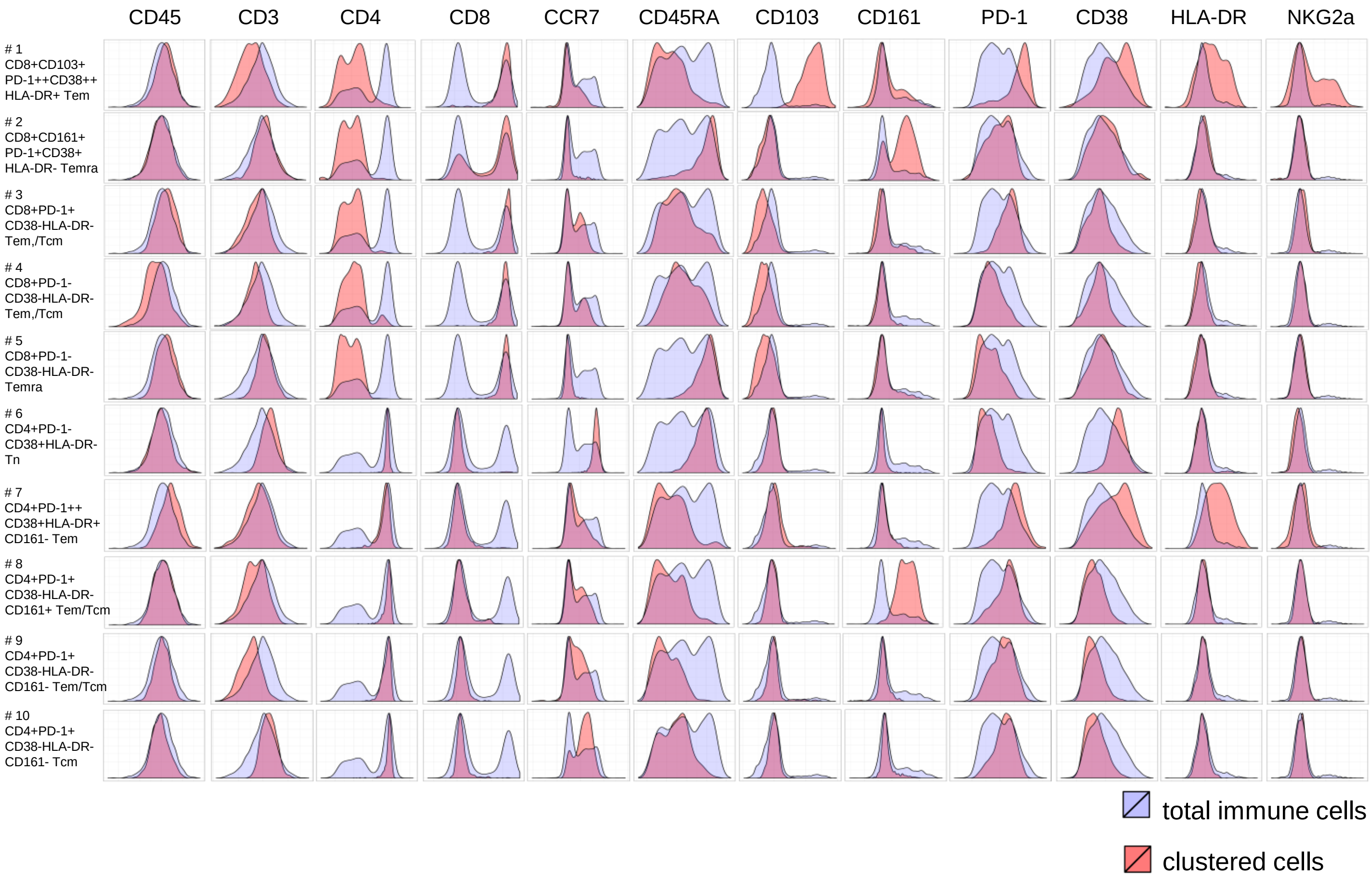

Additional file 11. Clustering analysis using CITRUS revealed 10 distinctive populations of CD4⁺ and CD8⁺ T cells. Automatic discovery of stratifying biological signatures to identify significantly different cell populations within tumor and blood samples was performed using the CITRUS algorithm (n=52, 11 healthy controls, 29 VSCC PBMC and 12 VSCC tumors). Analyses were done within the total CD3⁺ immune population for T cells. Phenotypic plots showing expression of the indicated cell surface markers for the identified CD8⁺ T-cell (# 1 to 5) and CD4⁺ T-cell (#6 to 10) populations. Expression of the designated markers is given in purple for the total analyzed immune cells and in red for the clustered cells (overlay).
