# Supplementary material for: High numbers of activated helper T cells are associated with better clinical outcome in early stage vulvar cancer, irrespective of HPV or p53 status
Source: J Immunother Cancer. 2019 Sep 3;7:236. doi: 10.1186/s40425-019-0712-z (PMC6724316; doi:10.1186/s40425-019-0712-z)
Supplement: Supplementary file 12 — Gating strategy for CD8+, non-Treg CD4+ and regulatory (Treg) T-cell populations. (PDF 263 kb) [file 40425_2019_712_MOESM12_ESM.pdf]

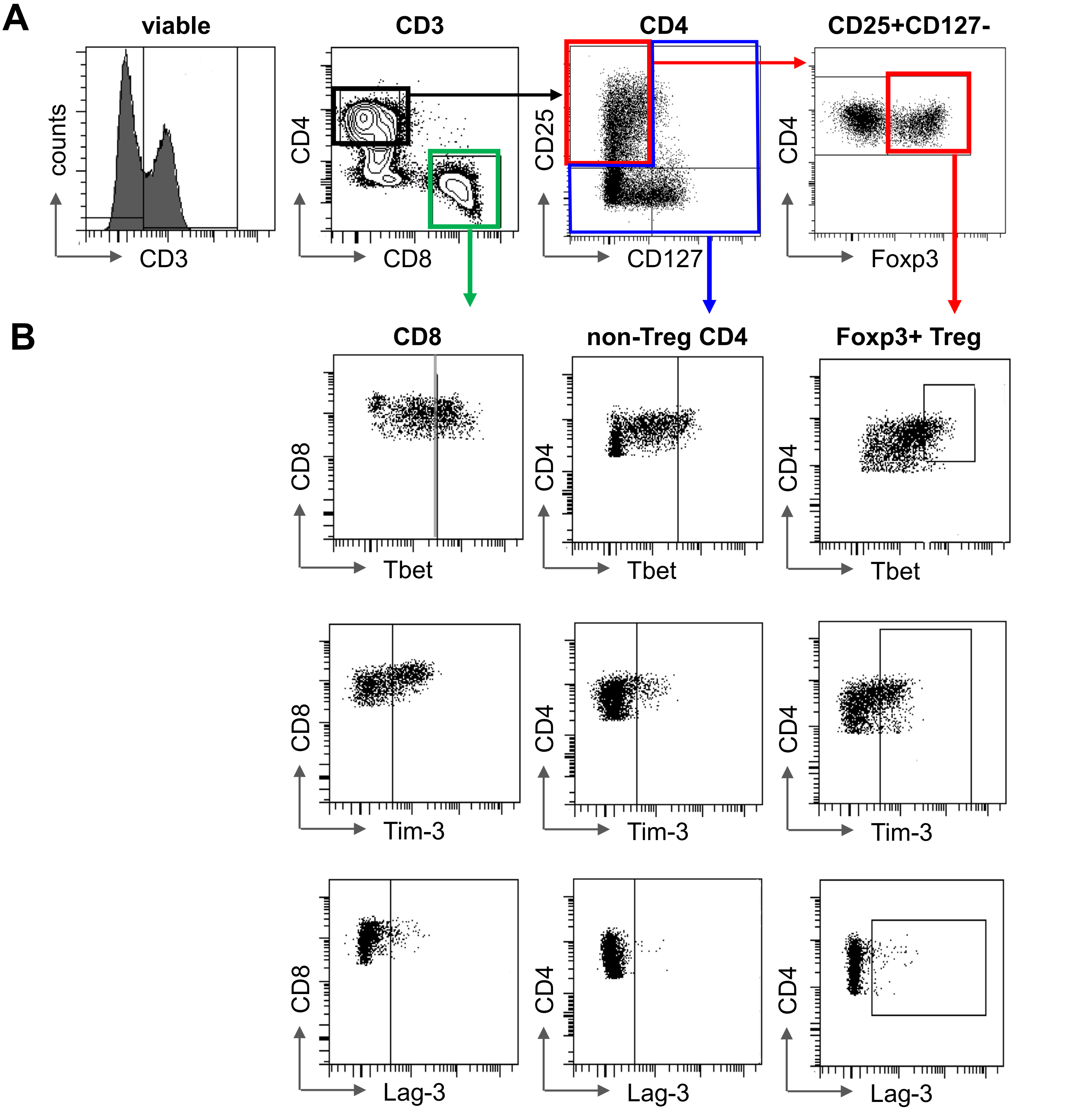

Additional file 12. Gating strategy for CD8⁺, non-Treg CD4⁺ and regulatory (Treg) T-cell populations. Characterization was done according to our previously publications on Treg and Tbet gating [31]. Gating strategy is depicted for a representative VSCC tumor sample. (**a**) Following doublet and dead cell exclusion, $CD3^+$ T cells were selected, which were further divided on the basis of CD4 and CD8 expression. Total $CD8^+$ T cells are gated in green. Within the $CD4^+$ T cells, CD25+CD127- cells (red) were selected for further Foxp3 gating ($Foxp3^+$ Tregs) and the non $CD25^+CD127^-$ $CD4^+$ T cells for further non-Treg CD4 gating (in blue). (**b**) Expression of the transcription factor Tbet (top panel), and the checkpoint molecules Tim-3 (middle panel) and Lag-3 (bottom panel) within $CD3^+CD8^+$ T cells (left), non-Treg $CD3^+CD4^+$ T cells (middle) and $CD4^+CD25^+CD127^-Foxp3^+$ T cells (right).
